# Estimation of patient-reported outcome measures based on features of knee joint muscle co-activation in advanced knee osteoarthritis

**DOI:** 10.1038/s41598-024-63266-7

**Published:** 2024-05-30

**Authors:** Iqram Hussain, Sung Eun Kim, Chiheon Kwon, Seo Kyung Hoon, Hee Chan Kim, Yunseo Ku, Du Hyun Ro

**Affiliations:** 1 Institute of Medical and Biological Engineering, Medical Research Center, Seoul National University College of Medicine, Seoul, 03080 Republic of Korea; 2 Department of Anesthesiology, Weill Cornell Medicine, Cornell University, New York, NY 10065 USA; 3 Department of Orthopedic Surgery, Seoul National University Hospital, Seoul National University College of Medicine, 101 Daehak-ro, Jongno-Gu, Seoul, 03080 Republic of Korea; 4 Medical Device Research Center, Department of Biomedical Research Institute, Chungnam National University Hospital, 282 Munhwa-ro, Jung-gu, Daejeon, 35015 Republic of Korea; 5 Department of Biomedical Engineering, College of Medicine, Chungnam National University, Daejeon, 35015 Republic of Korea; 6 Department of Biomedical Engineering, Seoul National University College of Medicine, Seoul, 03080 Republic of Korea; 7 Interdisciplinary Program in Bioengineering, Graduate School, Seoul National University, Seoul, 08826 Republic of Korea; 8 CONNECTEVE Co., Ltd, Seoul, 06224 Republic of Korea; 9 Innovative Medical Technology Research Institute, Seoul National University Hospital, Seoul, 03080 Republic of Korea

**Keywords:** Knee osteoarthritis, Electromyography, WOMAC, Machine-learning, Co-contraction index, Biomedical engineering, Medical research

## Abstract

Electromyography (EMG) is considered a potential predictive tool for the severity of knee osteoarthritis (OA) symptoms and functional outcomes. Patient-reported outcome measures (PROMs), such as the Western Ontario and McMaster Universities Osteoarthritis Index (WOMAC) and visual analog scale (VAS), are used to determine the severity of knee OA. We aim to investigate muscle activation and co-contraction patterns through EMG from the lower extremity muscles of patients with advanced knee OA patients and evaluate the effectiveness of an interpretable machine-learning model to estimate the severity of knee OA according to the WOMAC (pain, stiffness, and physical function) and VAS using EMG gait features. To explore neuromuscular gait patterns with knee OA severity, EMG from rectus femoris, medial hamstring, tibialis anterior, and gastrocnemius muscles were recorded from 84 patients diagnosed with advanced knee OA during ground walking. Muscle activation patterns and co-activation indices were calculated over the gait cycle for pairs of medial and lateral muscles. We utilized machine-learning regression models to estimate the severity of knee OA symptoms according to the PROMs using muscle activity and co-contraction features. Additionally, we utilized the Shapley Additive Explanations (SHAP) to interpret the contribution of the EMG features to the regression model for estimation of knee OA severity according to WOMAC and VAS. Muscle activity and co-contraction patterns varied according to the functional limitations associated with knee OA severity according to VAS and WOMAC. The coefficient of determination of the cross-validated regression model is 0.85 for estimating WOMAC, 0.82 for pain, 0.85 for stiffness, and 0.85 for physical function, as well as VAS scores, utilizing the gait features. SHAP explanation revealed that greater co-contraction of lower extremity muscles during the weight acceptance and swing phases indicated more severe knee OA. The identified muscle co-activation patterns may be utilized as objective candidate outcomes to better understand the severity of knee OA.

## Introduction

Knee osteoarthritis (OA) is a leading cause of discomfort and disability among elderly people. Moreover, sarcopenia results in reduced mobility, function, and quality of life and positively correlates with the severity of osteoarthritis^1^. Patient-reported outcome measures (PROMs) are assessment tools consisting of standardized questionnaires that allow patients to rate their level of pain, stiffness, difficulty with various activities of everyday living, and overall quality of life. Knee OA is characterized by progressive pain, stiffness, and functional impairment. The Western Ontario and McMaster University Osteoarthritis (WOMAC) index has been the gold standard for determining knee OA severity assessing pain, stiffness, and physical function^2^. The visual analog scale (VAS) is a 10-point scale used to assess pain intensity^3,4^. The results of PROMs are utilized to design treatment plans, evaluate treatment effectiveness, and understand disease prognosis.

Understanding the intrinsic mechanisms of knee OA-impaired biomechanics has led to the importance of understanding neuromuscular control given its apparent role in reinforcing knee OA^5,6^. Surface electromyography (EMG) revealed slight changes in muscle activation in mild to moderate knee OA patients compared to healthy controls during gait^7,8^, with larger differences between patients with more severe OA and asymptomatic individuals^9,10^. Changes in muscle activation patterns and increases in co-activation of muscle pairs are thought to occur in order to reduce medial compartment loading or improve joint stability. Studies investigating muscle activation patterns across groups with knee OA reported that higher muscle co-contractions were linked to the severity of knee OA^10–14^ and higher muscle forces^10,15^ to adjust for symptoms and joint instability. Information regarding the relationship between PROM-reported knee OA symptoms, functional outcomes, and neuromuscular and gait features is limited. Hence, investigating these measures can potentially contribute to objective biomarkers of knee OA symptoms and provide an understanding of the relationship between disease symptoms and gait.

Gait assessment is an effective method to identify functional limitations associated with knee OA and understand the relationship between disease symptoms and gait quality. Gait assessment utilizes 3D motion camera, force plates, pressure mats, and wearable inertial measurement sensors^16,17^. Although radiography is extensively utilized for diagnostics, a poor association was found between the radiographic grades utilized to diagnose knee OA progression and symptoms^18^. Previous studies have investigated the relationship between gait muscle activation and knee OA severity based on the Kellgren–Lawerence (KL) grades^5,19^ and classification of the KL grades using machine-learning approach based on key gait features^20^.

Machine learning (ML) and deep learning (DL) techniques have found extensive application across various domains, gait disorder, disease diagnosis, drug discovery, personalized treatment planning, and predictive analytics^21–24^. However, most ML models are considered “black boxes”, lacking easy interpretability by healthcare professionals^25^. To address this issue, explainable artificial intelligence (XAI) has emerged as an approach to enhance the interpretability and trustworthiness of ML models^22,23,25,26^, utilizing most popularly, local feature attributions^27^ and the Shapley value^28,29^.

We hypothesized that gait features on EMG would vary according to the severity of knee OA symptoms as indicated by the WOMAC and VAS scores. Although there are previous studies on the gait features of patients with knee OA, there was a subjective limitation in the degree of pain and discomfort in each patient^5,13^. Therefore, we characterized the key muscle activation and co-contraction features associated with the degree of knee OA severity according to the WOMAC and VAS scores. Moreover, we estimated the severity of knee OA using machine-learning methods and identified the neuromuscular features that contributed the most to estimating the self-reported symptoms (WOMAC pain and stiffness and VAS) and functional outcomes (WOMAC physical function) through XAI approaches based on Shapley value. The key contributions of this study are summarized as follows:

- We explored the EMG gait features of patients with knee OA who were scheduled to undergo total knee arthroplasty (TKA) and investigated features of muscle activation in mild, moderate, and severe knee OA according to the WOMAC and VAS.
- By leveraging machine learning and explainable AI techniques, our proposed interpretable knee osteoarthritis severity estimation model offers clinicians insights into the contribution of electromyography (EMG) gait features in knee OA severity estimation, enhancing clinical decision-making.

## Experiment and methodology

**Figure 1:**
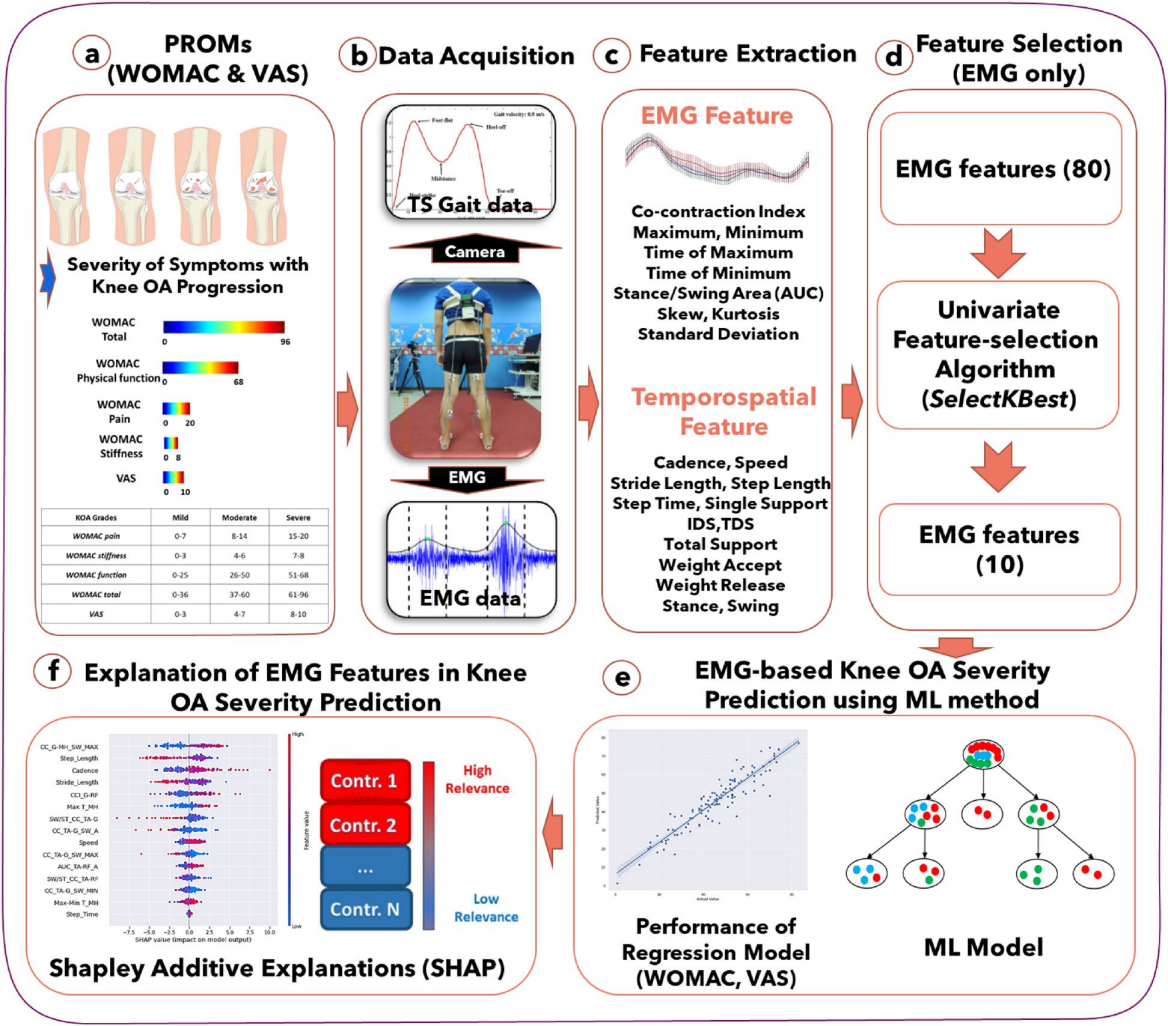
A methodological overview of electromyography (EMG)-based estimation of knee osteoarthritis (OA) severity using a machine-learning regression approach. (**a**) Description of patient-reported outcome measures (Western Ontario and McMaster Universities Osteoarthritis [WOMAC] and visual analog scale [VAS]). (**b**) Experimental scenario and data acquisition. (**c**) Feature extraction of EMG and temporospatial variables. (**d**) Feature selection method. (**e**) Estimation of WOMAC and VAS using machine-learning model. (**f**) Explanation of EMG feature contribution in ML prediction model using Shapley additive explanations (SHAP).

The methodology for estimating knee OA severity according to PROMs through EMG using a machine-learning regression approach is shown in Fig. 1. The experimental scenario and data acquisition process are detailed to provide context. Subsequently, the methodology involves the extraction of features from EMG and temporospatial variables. A specific feature selection method is applied to enhance the model's precision. The estimation of WOMAC and VAS is accomplished through a machine-learning model. Furthermore, the methodology incorporates SHAP to elucidate the contribution of EMG features, offering interpretability to the regression model's outcomes. Details are presented in the following subsections.

### Demographics of patients

The Institutional Review Board of Seoul National University Hospital (SNUH) has approved this study (IRB number: 1810-004-974). The procedures were in accordance with the ethical standards of the responsible committee on human experimentation and with the Helsinki Declaration of 1975, as revised in 2000 and 2008. All participants provided the written informed consents. Overall, 84 patients with knee OA (mean age: 69.81 ± 6.07 years, 87% women) were recruited for this study in the Laboratory of Human Motion Analysis, SNUH between January 2010 and December 2015. Nine Subjects were excluded due to a lack of PROMs; data from 75 subjects were investigated in this study. The participants were patients with advanced knee OA (Kellgren–Lawrence grade 3 or 4) and scheduled to undergo TKA. Patients were confirmed to have knee OA based on magnetic resonance imaging or computed tomography scans. Patients with any prior bone surgery in the lower extremities, spine disease, hip, or ankle arthritis detected on radiography, knee inflammatory or traumatic arthritis, cognitive impairment, or depression were excluded.

### Data acquisition

Raw EMG data were acquired using Telemyo 2400R G2 (Noraxon Inc., Scottsdale, AZ, USA) with Noraxon MyoResearch XP Master Edition 1.08.17 (Noraxon Inc., Scottsdale, AZ, USA) at a sampling rate of 1500 Hz. The system consists of a Telemyo 2400R G2 receiver, Telemyo 2400 T G2 transmitter with eight channels, pre-amplified lead wires, and disposable electrodes. The acquisition of motion data was done using a 3D optical motion capture system (Motion Analysis Corp., Santa Rosa, CA, USA) at 120 Hz sampling rate. Gait phase information was obtained using two force plates embedded in the floor over the 9-m walkway. An operator placed reflective markers and EMG electrodes on the subjects based on the Helen Hayes set. The subjects walked for a few minutes to familiarize themselves with the setting. EMG data was recorded from four major lower extremity muscles of both lower limbs: rectus femoris (RF), medial hamstring (MH), tibialis anterior (TA), and gastrocnemius (G). EMG data were coupled and recorded by the motion capture program Cortex 8.1.0 (Motion Analysis Co., Santa Rosa CA). These muscle selections were made due to their primary roles in supportive and propelling forces during the gait process^30^. The motion and EMG data for each joint were averaged after five or six trials of the entire walk^31^.

### Pre-processing

Post-processing of EMG raw data was performed using Orthotrak 6.6.4 (Motion Analysis Co., Santa Rosa, CA, USA). EMG raw signals were initially filtered with a bandpass filter (20–450 Hz). Then, we applied a band-stop filter to remove 60 Hz AC noise from the EMG signal. Moreover, we calculated the signal-to-noise ratio (SNR) for each signal to remove motion artifacts by comparing raw EMG signals with undisturbed EMG signals obtained immediately after muscle contraction. EMG epochs with an SNR below 18 dB, indicating inadequate signal quality, were excluded from the dataset^32,33^. Then, all kinematic and EMG signals were full-wave bi-directional rectified and filtered using a second-order low-pass Butterworth filter at a cutoff frequency of 6 Hz to remove noise^34,35^. The resulting waveform was then dilated using a linear interpolation scheme to match the sample rate of the video and to create a linear gait envelope for each muscle. The EMG envelope was obtained by normalizing EMG to 100% of the gait cycle based on temporal markers (heel strikes and toe-offs) and subdivided into the stance phase and swing phase^36^.

### Categorization of knee OA severity using PROMs

Categorization of PROMs-based knee osteoarthritis severity has adapted to the investigation of the correlation between feature trends and the severity of knee OA symptoms^17,37,38^. The WOMAC index is a self-administered PROM tool that includes 24 items containing pain, stiffness, and physical function, with each question scored from 0 to 4 representing the severity of symptoms^2^. The VAS is a PROM tool with a 10-point horizontal line for measurement of the severity of pain and discomfort faced by patients with knee osteoarthritis^3^. All participants answered the WOMAC Questionnaires with three subscales^39^. The possible score ranges from 0 to 20, 0 to 8, 0 to 68, and 0 to 96 for pain, stiffness, physical function, and WOMAC total score, respectively. The WOMAC score categorizes the severity of knee osteoarthritis (OA) into different levels (none, mild, moderate, severe, and extreme) based on the PROM obtained in pain, stiffness, and physical function dimensions^2^. We classified the severity of knee OA based on the WOMAC score into three severity groups: mild, moderate, and severe to investigate the correlation between feature trends and the severity of knee OA symptoms as proposed in our previous study^17^. To establish these categories, we determined cut-off points by selecting the midpoints of the 4-point WOMAC score between mild to moderate and moderate to severe, and then multiplication by 24, the number of WOMAC questionaries. The cut-off points are 1.5, the midpoint between mild and moderate, and 2.5, the midpoint between moderate and severe. Subsequently, WOMAC scores below 36, between 36 and 60, and above 60 were categorized as mild, moderate, and severe, respectively^17^. We applied a similar methodology to categorize WOMAC pain, WOMAC stiffness, WOMAC physical function, and VAS scores into three distinct classes, as illustrated in Table 1 and Table 2.

**Table 1:** Characteristics of the severity of knee osteoarthritis (OA) according to WOMAC and VAS, and its categorization in the corresponding lower extremities.

Features	Mild (n = 36)	Moderate (n = 83)	Severe (n = 31)	*p*-value
WOMAC total	24.81 (7.84)	48.05 (6.16)	72.10 (9.83)	< 0.001
WOMAC physical function	17.69 (6.34)	35.27 (4.79)	52.35 (6.55)	< 0.001
WOMAC pain	5.08 (2.55)	9.39 (2.66)	13.55 (3.78)	< 0.001
WOMAC Stiffness	2.03 (1.42)	3.40 (1.87)	6.19 (1.30)	< 0.001
VAS	1.76 (1.1)	5.95 (1.14)	8.76 (0.82)	< 0.001

WOMAC: Western Ontario and McMaster Universities Osteoarthritis index; VAS: visual analog scale. n refers to individual lower extremities.

**Table 2:** Patient demographic of the severity of knee osteoarthritis (OA) according to WOMAC and VAS.

Features	Mild (n = 18)	Moderate (n = 42)	Severe (n = 15)	*p*-value
Age	70.08 (7.12)	69.43 (6.39)	70.52 (3.40)	–

### Gait feature extraction

Statistical features were extracted from the EMG-derived normalized gait cycle waveform. The features included mostly fiducial features of the EMG gait envelope not limited to the average, maximum, and minimum amplitude of the EMG waveform, maximum and minimum peak latency, and the co-contraction index (CCI) of muscle pairs over the full gait cycle, stance, and swing phase. Additionally, CCI was calculated over the gait cycle by using Eq. (1), developed by Rudolph et al.^40,41^, for the following muscle sets: TA-G, RF-MH, TA-MH, and RF-G.

1 $$\begin{array}{*{20}c} {CCI\left( t \right) = \frac{{EMG_{L} \left( t \right)}}{{EMG_{H} \left( t \right)}}\left( {EMG_{L} \left( t \right) + EMG_{H} \left( t \right)} \right)} \\ \end{array}$$

2 $$\begin{array}{*{20}c} {CCI = \mathop \sum \limits_{{t = t_{1} }}^{{t = t_{2} }} {\raise0.7ex\hbox{${\left[ {CCI\left( t \right)} \right]}$} \!\mathord{\left/ {\vphantom {{\left[ {CCI\left( t \right)} \right]} {\left( {t_{2} - t_{1} } \right)}}}\right.\kern-0pt} \!\lower0.7ex\hbox{${\left( {t_{2} - t_{1} } \right)}$}}} \\ \end{array}$$

This equation allows for the quantification of co-contraction between two muscles during a given time frame. “EMG_L_” and “EMG_H_” denote the antagonist and agonist EMG data, respectively, resampled to 101 normalized time (t) points (0–100% of gait cycle at 1% increments). t_1_ and t_2_ are the starting and ending nodes, respectively, of the full cycle, stance phase, and swing phase. Moreover, all temporospatial variables, not limited to speed, step length, stride length, and cadence, were extracted over the gait cycle using motion camera and force plate data.

### Statistical analysis

Statistical analysis was performed using SPSS 26 software (IBM, Armonk, NY, USA). One-way analysis of variance (ANOVA) was performed to identify the significant differences (significance level of 0.001) of features. Student’s t-test was utilized to analyze class differences between each severity group based on each subscale of the WOMAC and VAS.

### Machine-learning approach

Machine learning regression models have been utilized to estimate the severity of knee OA symptoms according to the PROMs using EMG gait features. Scikit-learn ver. 1.3.1 was used for machine learning analysis using Python 3.11^42^. ML analysis was conducted in Google Colab, leveraging its 16 GB RAM and 2-core Intel Xeon Processor. Our developed ML model included a range of functionalities such as feature selection, ML regression model, and model explainability. To enhance model interpretability, we utilized the SHAP library, assigning weights to features for better regression insights^25^.

#### Feature selection and dimensionality reduction

SelectKBest is a univariate feature selection that works by selecting the best features based on univariate statistical tests for reducting feature set. We utilized SelectKBest, available in Scikit-learn^42^, to select higher correlated EMG features according to the k highest scores.

#### ML regression model for knee OA severity prediction

Various machine learning models, including multiple linear regression, random forest (RForest), ridge regression, lasso regression, and support vector regression, have been employed in the development of regression models. RForest is an ensemble learning method, constructing multiple decision trees through different data subsets, and voting on the results of multiple decision trees to get the output of the random forest^43^. RForest regression analysis was performed to estimate the WOMAC index, examine its relationship with WOMAC key features, and observe the feasibility of the prediction model. A training dataset and a testing dataset have been created by allocating 80% and 20% of the dataset, respectively. The testing dataset is intended for evaluating the performance of the developed model on previously unseen data. We validated the trained models using k-fold cross-validation (CV). The model was analyzed by observing the root mean square error (RMSE) and correlation between actual and estimated WOMAC score and its sub-scores.

### ML model interpretation using explainable artificial intelligence (XAI)

Shapley Additive Explanations (SHAP) is an explainable AI (XAI) method based on game theory that can make explanations for local and global ML models^25^. To produce an interpretable model, SHAP uses an Shapley values based additive feature attribution method, defining an output model as a linear addition of input variables. SHAP can identify the contribution of each input feature and can help interpret the model globally as well as locally. SHAP values can be approximated by various methods, such as Kernel SHAP, Deep SHAP, and Tree SHAP. Among these methods, a Tree SHAP for tree-based RForest machine learning models was used in this study to make an explanation of EMG and TS features for estimating WOMAC and VAS. The mathematical expression of the Shapley value, *ϕ*_*i*_(*x*) of feature *i* in predicting the output:

3 $$\begin{array}{*{20}c} {\phi_{i} \left( x \right) = \mathop \sum \limits_{{{\raise0.5ex\hbox{$\scriptstyle {S \subseteq N}$} \kern-0.1em/\kern-0.15em \lower0.25ex\hbox{$\scriptstyle {\left\{ i \right\}}$}}}} \frac{{\left| S \right|!\left( {\left| N \right| - \left| S \right| - 1} \right)!}}{\left| N \right|!}\left( {f\left( {x_{s} \cup \left\{ i \right\}} \right) - f\left( {x_{s} } \right)} \right)} \\ \end{array}$$

where *N* is the set of all features; *S* is a subset of features excluding *i*; *x*_*S*_ represents the instance *x* with only features in subset *S*; *f*(*x*_*S*_) is the model's prediction for instance *x*_*S*_; *f*(*x*_*S*_* ∪ *{*i*}) is the model's prediction for instance *x* with features in subset *S* plus feature *i*. SHAP^44^, a feature attribution approach using Shapley value, was employed for interpreting the contributions of EMG features in the knee OA severity prediction model. Interventional TreeSHAP^45^ is ideal for explaining Shapley values in tree-based ML models like decision trees, random forests, and gradient-boosting models. TreeSHAP stands out for being non-trivial, bias-free, and free from variance issues^25^.

### Ethics declarations

The Institutional Review Board of Seoul National University Hospital (SNUH) has approved this study (IRB number: 1810-004-974). The study was conducted in accordance with the ethical standards of the Helsinki Declaration. All participants provided written informed consent.

### Consent to participate/consent to publish

All participants provided written informed consent for participation and publication.

## Results

### Muscle activation patterns

**Figure 2:**
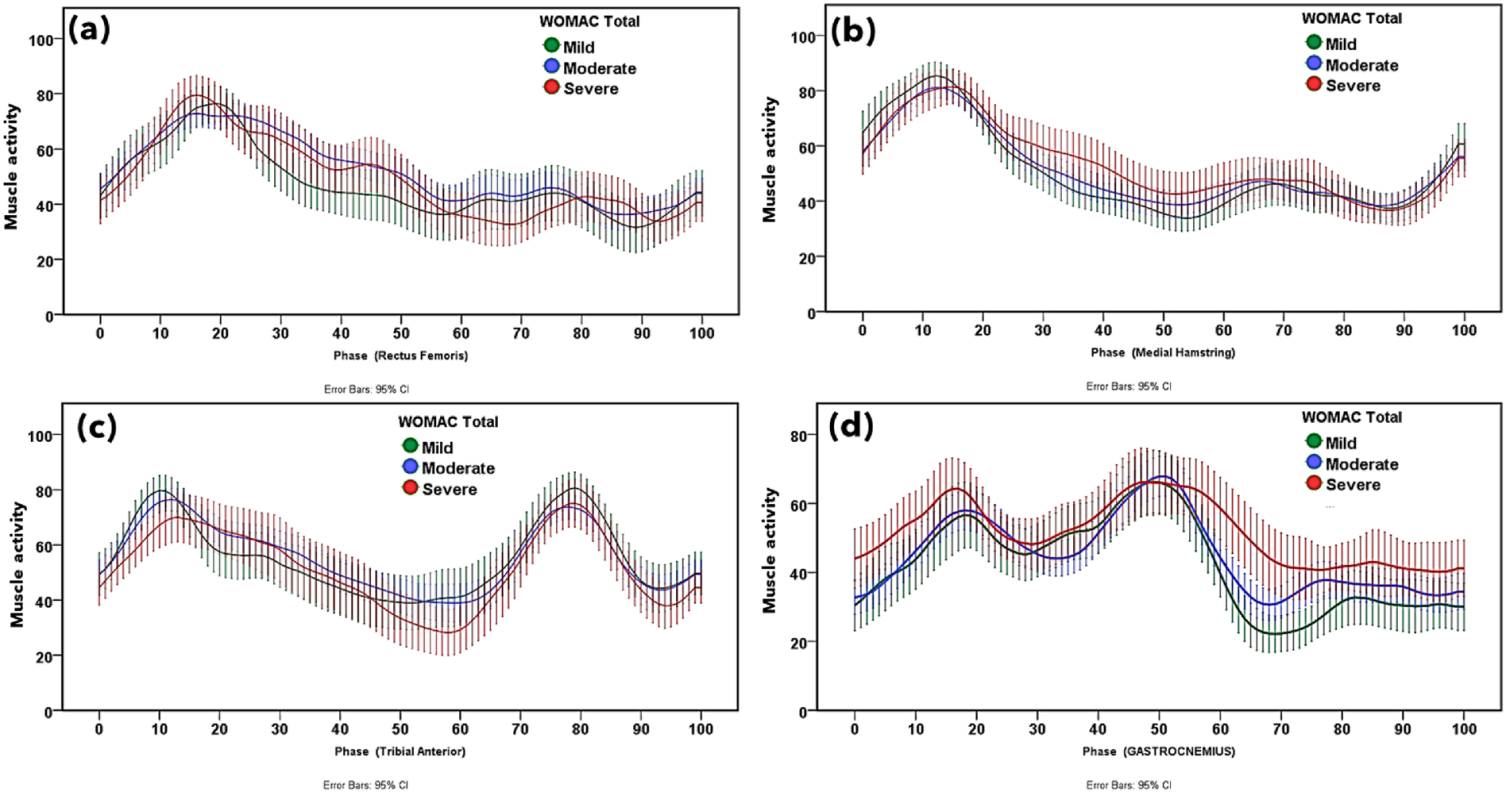
Muscle activation pattern of the (**a**) rectus femoris, (**b**) medial hamstring, (**c**) tibialis anterior, and (**d**) gastrocnemius muscles in mild, moderate and severe knee osteoarthritis according to the Western Ontario and McMaster Universities Osteoarthritis (WOMAC) score. The error bar shows 95% confidence interval.

Normalized EMG patterns of the RF, MH, TA, and G muscles based on knee OA severity according to the WOMAC scores are shown in Fig. 2. Moreover, normalized EMG patterns of these muscles for the WOMAC subcategories and VAS are displayed in Figs. S1, S2, S3 and S4. Group ensemble-averaged waveforms for the RF, MH, TA, and G muscles qualitatively illustrated the gait cycle with functional outcomes associated with knee OA severity based on the WOMAC.

Gastrocnemii activity increased with an increase in the WOMAC total especially in the weight acceptance and swing phases, similar to those for the WOMAC subcategories. Higher G activity was observed with an increase in patient-reported pain during the heel-strike phase according to the WOMAC pain score. Moreover, greater G activity was observed with worsening WOMAC physical function during the heel-strike and swing phases. Additionally, G activity was dominant throughout the gait cycle with higher knee stiffness. No variation in G activity was observed when based on the VAS score.

Higher MH activity was observed with worse symptoms according to the WOMAC scores. Progressive MH activity was observed from midstance to toe-off in knees with higher stiffness. No discriminative trend was observed in the MH activity with changes in knee OA symptom severity according to the WOMAC pain and function scores. Moreover, patients with higher VAS scores showed increased MH activity from midstance to toe-off during the stance phase. The RF activity pattern did not show any discriminative trend with WOMAC and its subcategories. Patients with higher VAS scores showed higher RF activity during midstance to toe-off during the stance phase. No discriminative trend was found between TA activity and symptoms and functional outcomes as reported in the VAS and WOMAC.

### Muscle co-contraction index

**Figure 3:**
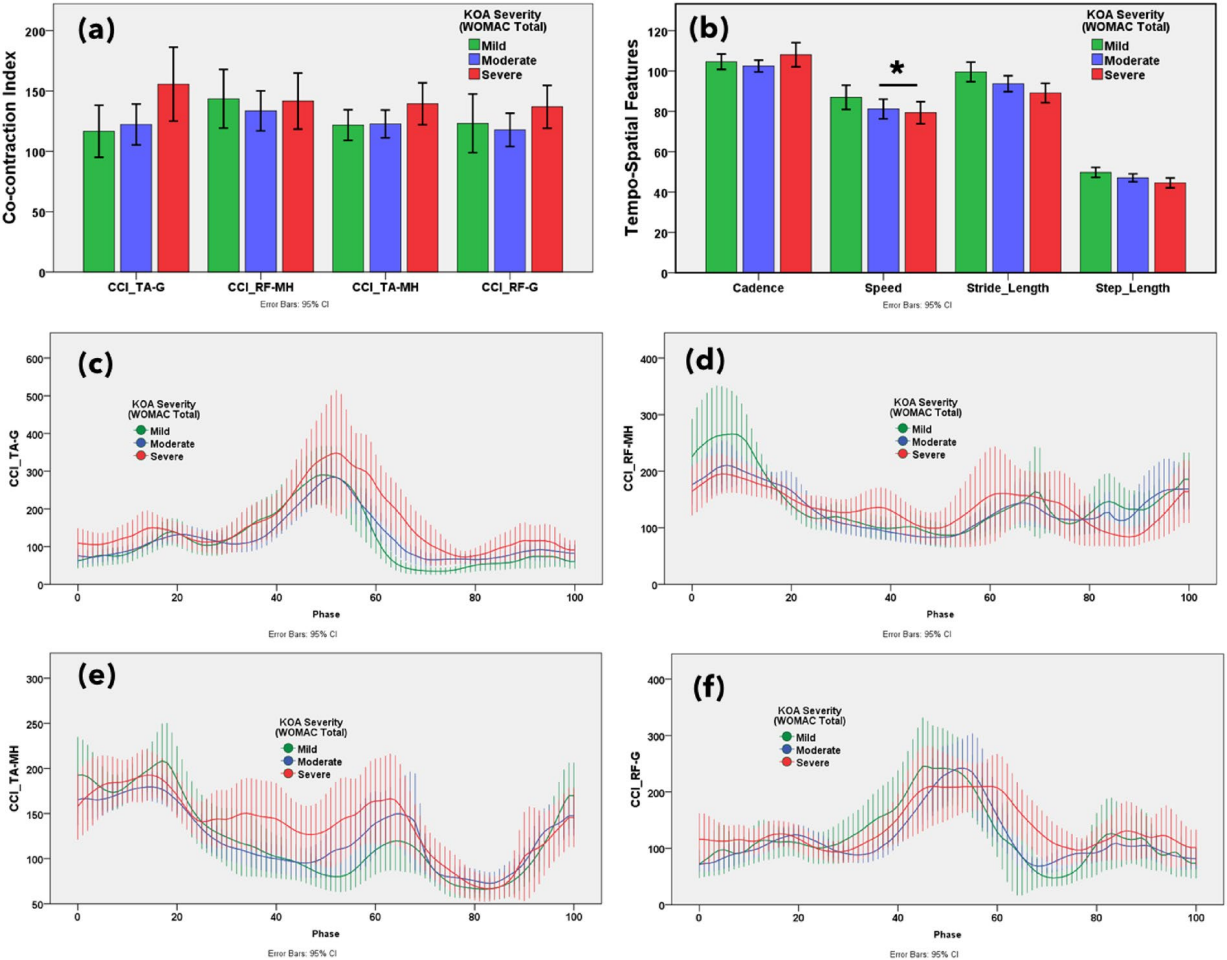
(**a**) Statistical distribution of the co-contraction index of the TA–G, RF–MH, TA–MH, and RF–G muscle pairs. (**b**) Temporospatial features in mild, moderate, and severe knee osteoarthritis. Severity based on the Western Ontario and McMaster Universities Osteoarthritis (WOMAC) total score. Muscle co-activation pattern of (**c**) TA–G, (**d**) RF–MH, (**e**) TA–MH, and (**f**) RF–G muscle pairs in mild, moderate, and severe knee osteoarthritis. Severity based on the WOMAC total score. *p < 0.05 indicates significant difference. The error bar shows 95% confidence interval.

**Figure 4:**
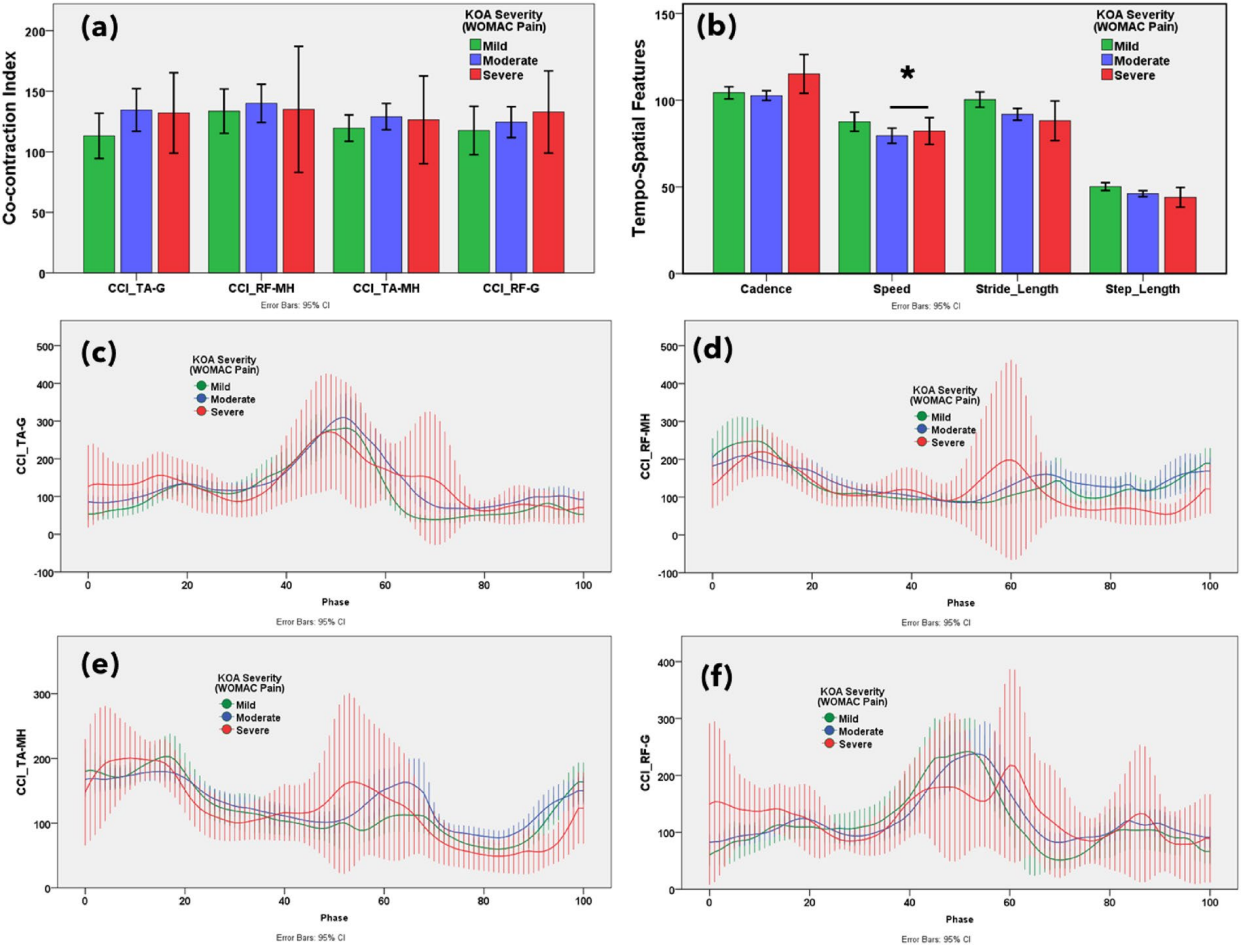
(**a**) Statistical distribution of the co-contraction index of the TA–G, RF–MH, TA–MH, and RF–G muscle pairs. (**b**) Temporospatial features in mild, moderate, and severe knee osteoarthritis. Severity based on the Western Ontario and McMaster Universities Osteoarthritis (WOMAC) pain subscore. Muscle co-activation pattern of (**c**) TA–G, (**d**) RF–MH, (**e**) TA–MH, and (**f**) RF–G pairs in mild, moderate, and severe knee osteoarthritis. Severity based on the WOMAC pain subscore. *p < 0.05 indicates significant difference. The error bar shows 95% confidence interval.

**Figure 5:**
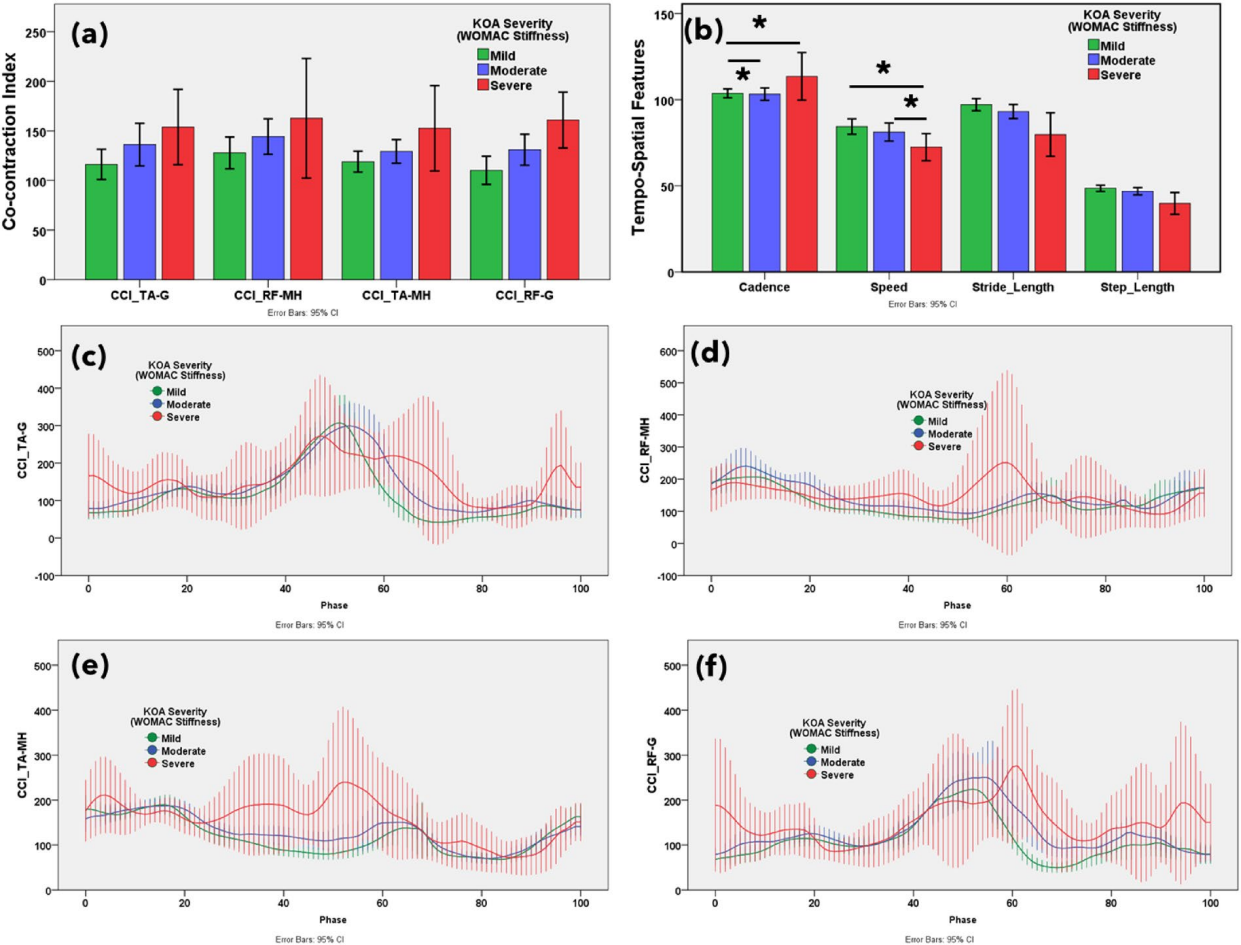
(**a**) Statistical distribution of the co-contraction index of the TA–G, RF–MH, TA–MH, and RF–G muscle pairs. (**b**) Temporospatial features in mild, moderate, and severe knee osteoarthritis. Severity based on the Western Ontario and McMaster Universities Osteoarthritis (WOMAC) stiffness subscore. Muscle co-activation pattern of (**c**) TA–G, (**d**) RF–MH, (**e**) TA–MH, and (**f**) RF–G pairs in mild, moderate, and severe knee osteoarthritis. Severity based on the WOMAC stiffness subscore. *p < 0.05 indicates significant difference. The error bar shows 95% confidence interval.

**Figure 6:**
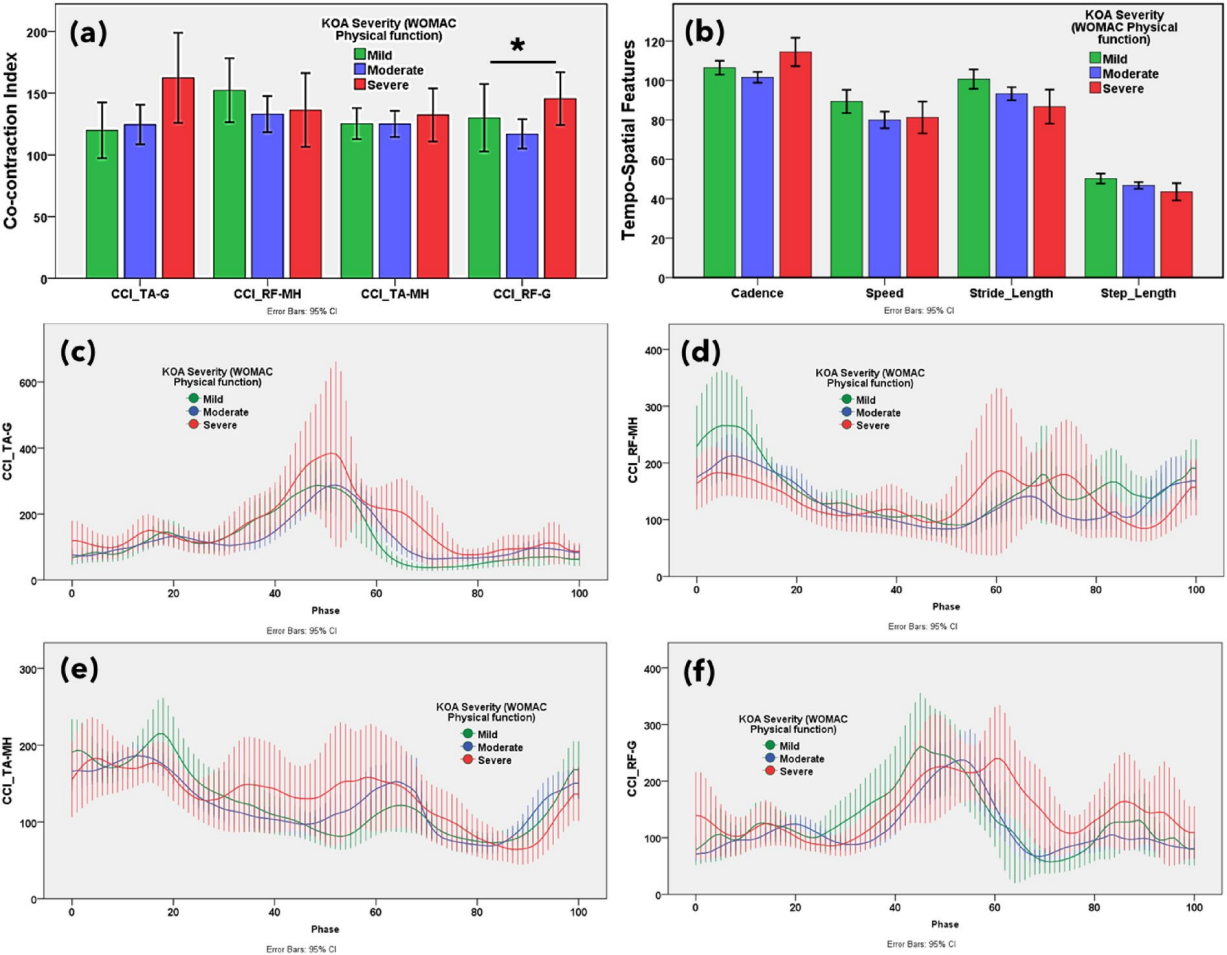
(**a**) Statistical distribution of the co-contraction index of the TA–G, RF–MH, TA–MH, and RF–G muscle pairs. (**b**) Temporospatial features in mild, moderate, and severe knee osteoarthritis. Severity based on the Western Ontario and McMaster Universities Osteoarthritis (WOMAC) physical function subscore. Muscle co-activation pattern of (**c**) TA–G, (**d**) RF–MH, (**e**) TA–MH, and (**f**) RF–G pairs in mild, moderate, and severe knee osteoarthritis. Severity based on the WOMAC physical function subscore. *p < 0.05 indicates significant difference. The error bar shows 95% confidence interval.

The muscle co-contraction index between antagonist and agonist muscles on functional outcomes associated with knee OA severity according to WOMAC and VAS scores are reported in Figs. 3a, 4a, 5a, 6a and S5a, respectively. Additionally, the muscle co-contraction patterns of muscle pairs according to the WOMAC and VAS reported functional outcomes are shown in Figs. 3, 4, 5, 6 and S5, respectively.

The CCI of TA–G and TA–MH progressively increased with increasing severity of symptoms according to the WOMAC scores. Additionally, the CCI of the RF–G increased from mild to severe based on the PROMs. This suggests that the severity of knee OA alters muscle co-activation. A higher CCI for TA–G and RF–G indicated higher WOMAC pain scores. The CCI of all muscle pairs (TA–G, RF–MH, TA–MH, and RF–G) showed an increasing trend with higher WOMAC stiffness scores. A higher CCI for TA–G indicates higher WOMAC physical function scores. The VAS score showed mixed results with respect to the CCI. Notably, muscle co-activation peaked in the pre-swing phase (approximately 50–60% of the gait cycle). In the pre-swing phase, patients with severe knee OA showed greater muscle activation. Higher co-contraction of antagonist and agonist muscle pairs indicated more severe knee OA according to the WOMAC and VAS scores.

### Temporospatial features

The gait temporospatial features according to the functional outcomes, reported on WOMAC and VAS, are illustrated in Figs. 3b, 4b, 5b, 6b, and S5b, respectively. Speed, stride, and step length tended to decrease with an increase in the WOMAC and VAS scores. Cadence (number of steps per unit time) was not associated with changes in knee OA severity.

### Estimation of knee OA severity using ML regression approach

#### Feature selection and hyperparameter optimization

The feature selection method was utilized to reduce the number of input features followed by training of the regression models with hyperparameter tuning and cross-validation. We utilized SelectKBest^42^ to select the 15 EMG features with the k-highest scores in the ML regression modeling. We optimized hyperparameter tuning through max_depth criteria to achieve the best value for co-efficient of determination (R2). The maximum tree depths of the RForest model were 9, 9, 8, 9, and 9 for WOMAC total, pain, stiffness, and physical function, and VAS, respectively.

#### Performance of knee OA severity estimation model

**Figure 7:**
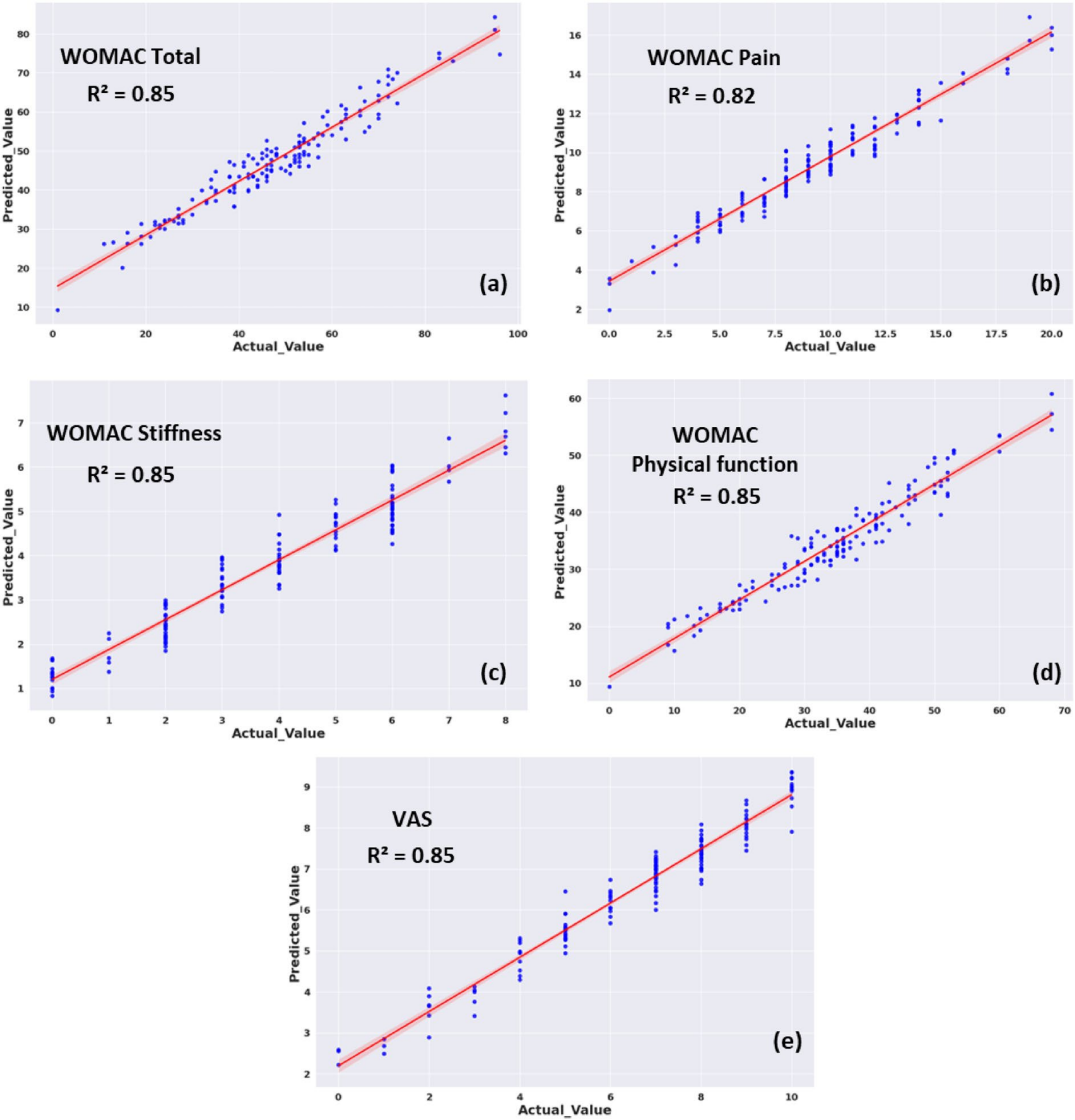
Performance outcomes of a machine-learning regression model in estimating the severity of functional limitations based on Patient-Reported Outcome Measures (PROMs) using an RForest regression algorithm with EMG features. The figure depicts the regression line (red) and scatter plot (blue) showcasing the machine learning predictions versus actual PROMs score for (**a**) WOMAC total, (**b**) WOMAC pain, (**c**) WOMAC stiffness, (**d**) WOMAC physical function, and (**e**) VAS scores. CCI: co-contraction index, RF: rectus femoris, MH: medial hamstring, TA: tibialis anterior, G: gastrocnemius, A: Average, MAX: maximum, MIN: minimum, ST: stance phase, SW: swing phase, T: time latency, AUC: area under the curve; VAS, visual analog scale; WOMAC, Western Ontario and McMaster Universities Osteoarthritis index; EMG, electromyography.

The regression model based on RForest algorithms demonstrated superior performance in accurately estimating WOMAC and VAS scores. The prediction performance of k-fold (k = 5) CV RForest regression modeling using EMG variables for WOMAC and its subcategories, and VAS are shown in Fig. 7. The R2 of this CV regression model was 0.85, 0.85, 0.85, 0.86, and 0.84 for WOMAC total, pain, stiffness, physical function, and VAS, respectively. Regression results for estimating WOMAC Total score using a RForest algorithm based on EMG features with testing dataset are shown in Fig. S6. Figure S8 illustrates the predictive performance of knee OA using RForest regression modeling, incorporating both EMG and temporospatial variables, across WOMAC and its subcategories, as well as VAS.

### Interpreting EMG-based ML model for predicting knee OA severity

Figures S7 and S8 display the SHAP feature importance and summary plots by showing the 10 most important EMG features as evaluated by SHAP and its effects on estimating the WOMAC and VAS scores. Figure S9 presents the feature attribution summary plot, highlighting the most impactful EMG and temporospatial features and their roles determined by the Shapley value.

#### EMG feature contribution in knee OA severity prediction model

The SHAP feature importance plot reports the mean SHAP value, which describes the relative importance of each EMG feature for estimating functional limitations according to WOMAC and VAS (Fig. S7). Nomenclatures of features are given in the supplementary section.

Figure S7a shows the SHAP importance plots for the estimation of the WOMAC total. Single normalized EMG features of G and MH along with the co-contraction of TA–G and RF–G are among the most important gait features to estimate the WOMAC total score. In Fig. S7b, a single normalized MH EMG feature along with co-contraction of TA–MH and TA–G are among the most important gait features to estimate the WOMAC pain score. In Fig. S7c, a single normalized G EMG feature along with co-contraction features of RF–G and TA–H are the most important gait features to estimate the WOMAC stiffness score. In Fig. S7d, a single normalized G EMG feature along with co-contraction features are the most important gait features to estimate the WOMAC physical function score. In Fig. S7e, single normalized EMG features of RF and MH muscle along with co-contraction features of TA–G and TA–MH are the most important gait features to estimate the limitations of functional outcomes according to VAS score.

#### EMG feature trends in knee OA severity prediction model

**Figure 8:**
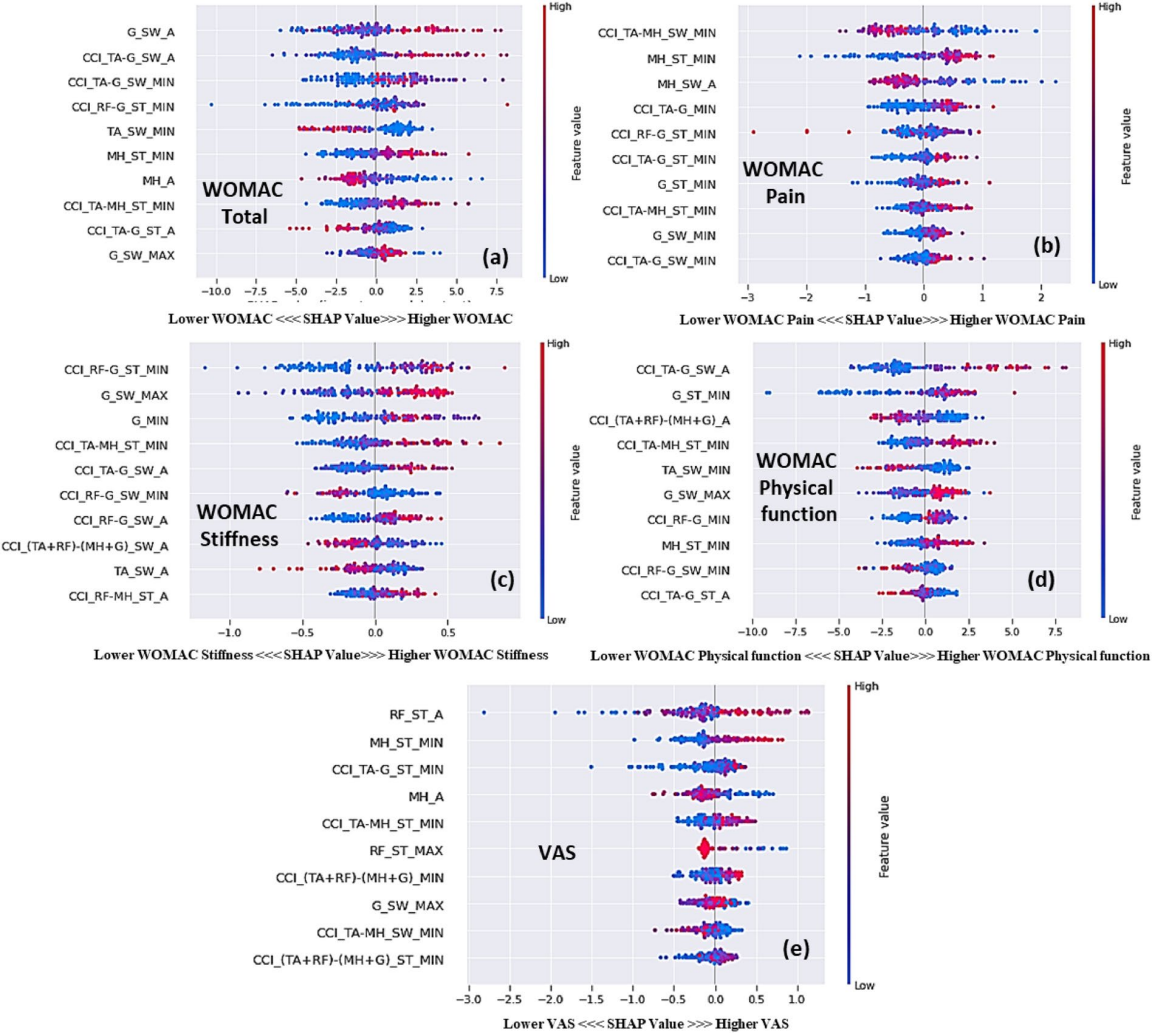
Interpreting the contributions of EMG features to estimate the knee OA severity, as demonstrated in the SHAP Summary plot, with a focus on (**a**) WOMAC total, (**b**) WOMAC pain, (**c**) WOMAC stiffness, (**d**) WOMAC physical function, and (**e**) VAS scores, ranked by importance. CCI: CCI: co-contraction index, RF: rectus femoris, MH: medial hamstring, TA: tibialis anterior, G: gastrocnemius, A: Average, MAX: maximum, MIN: minimum, ST: stance phase, SW: swing phase, T: time latency, AUC: area under the curve; VAS: visual analog scale; WOMAC: Western Ontario and McMaster Universities Osteoarthritis index; EMG: electromyography.

The range and distribution of the impacts of the EMG variables on the WOMAC and VAS scores were revealed through summary plots (Fig. 8). Each point on the summary plot is a SHAP value representing the input variables and an instance. Features with higher SHAP values indicate higher contributions to predicting functional limitations associated with knee OA severity in an ML model.

Higher values for *G_SW_A, CCI_TA-G_SW_A, MH_ST_MIN*, and *CCI_TA-MH_ST_MIN,* which have higher positive SHAP values, led to a higher WOMAC total score (Fig. 8a). This is compatible with previous results indicating that patients with higher WOMAC scores have increased co-contraction of the tibialis anterior with the gastrocnemius and medial hamstrings due to discomfort when walking. Higher values for *MH_ST_MIN, CCI_TA-G_MIN,* and *G_ST_MIN* indicated higher WOMAC pain scores. Similarly, lower values of *CCI_TA-MH_SW_MIN* and *MH_SW_A* indicated worse pain (Fig. 8b). Likewise, patients with higher WOMAC pain scores had a higher value at the troughs of MH and G in the stance phase (Fig. S1). Moreover, patients with higher WOMAC pain scores had higher co-contraction of TA–G due to pain when walking. Higher values for *CCI_RF-G_ST_MIN, CCI_TA-MH_ST_MIN,* and *CCI_TA-G_SW_A*, which represent higher positive SHAP values, indicated higher WOMAC stiffness scores (Fig. 8c). Similarly, patients with higher WOMAC stiffness scores had a higher co-contraction of the TA–G and RF–G in the swing phase and the TA–MH in the stance phase to counteract knee stiffness (Figs. 5 and S2). Higher co-contraction of TA–*G* in swing phase and lower *TA_SW_MIN* led to higher WOMAC physical function scores (Fig. 8d). Patients with higher WOMAC stiffness scores had a higher G amplitude in the weight acceptance and swing phases and a higher co-contraction of TA–G when walking due to impairment in physical function (Figs. 6 and S3). Higher co-contraction of TA–G and TA–MH*,* and lower values for *MH_A* tended to be higher pain outcomes (Fig. 8e). Likewise, patients with higher VAS scores had higher co-contraction of the tibialis anterior with the gastrocnemius and medial hamstrings due to pain during walking and stabilization (Fig. S5).

## Discussion

In this study, PROMs (WOMAC and VAS) were investigated in patients with severe knee OR who were scheduled to undergo TKA. Additionally, we explored the EMG features of the knee according to the PROMs. Although there have been studies on the EMG patterns of knees with and without OA, few studies have evaluated EMG studies while considering the degree of pain, stiffness, and physical function of patients with knee OA pre-TKA. Hence, we investigated changes in neuromuscular features according to the degree of pain, stiffness, and physical function and developed a regression model to estimate the severity of knee OA using EMG features.

According to this study, patients with higher WOMAC and VAS scores had relatively greater G and MH activity. An increase in knee OA severity led to increased muscle amplitude of the G, MH, and RF muscles, similar to our findings^5^. Patients with knee OA had greater muscle activation during the load acceptance phase, which may represent an attempt to induce muscle activation later in the gait cycle^11^. It may be logical that patients with worse knee OA had greater muscle action in the late swing phase to improve muscle function as the body weight is received.

All gastrocnemius waveform features changed progressively with increasing OA severity. Gastrocnemius muscle activity increased with an increase in the WOMAC total score especially in the swing and weight acceptance phases, suggesting an attempt to reduce the impact of heel strikes. These changes are consistent with responses to increased OA severity and are aimed at reducing the joint contact force and increasing the active stiffness in the early stance^5^. No significant changes were observed in the TA regardless of OA severity.

Less prominent changes in the medial hamstrings were observed with higher knee OA severity, mainly during midstance phase. With the worsening of OA, active stiffness was necessary in the lateral and medial compartments of the knee. The hamstring muscle responds to higher tensile strain on the tibiofemoral joint structures, delivering the adduction moment^46^. The stability of the medial compartment due to the interaction between joint gap shrinking and osteophytosis, contributes to the reduction of medial compartment loads^8,47^. The activity of the MH and RF muscles increases during the midstance/toe-off (push-off) phase, suggesting that patients used these muscles in the push-off phase to increase gait stability^48^.

CCI gives a measure of the comparative activation pattern of muscle pairs during phases of the gait cycle. Based on self-reported measures, CCIs increase progressively with worsening knee OA. CCIs for severe knee OA were higher than those for mild and moderate knee OA based on the WOMAC and VAS scores. The increased CCI for TA–G in the severe OA group was based on increased G activity whereas no clear differences were observed in TA activation among groups during the early stance and swing phases. A higher CCI for the G was observed during the early stance phase in individuals with knee OA^49^. In this study, the CCI for TA–G and RF–G gradually increased in knees with severe OA compared to those with mild or moderate OA. Increased MH coactivation improves knee joint stability^50^. In this study, knee joint stiffness best correlated with the CCI, which is in agreement with previous literature indicating that muscle co-contraction produces joint stiffness to enhance stability and precision during limb movement at the cost of higher energy^51^. Knee OA results in knee stiffness during weight acceptance, resulting in greater co-contraction of the knee flexors and extensors^11^. The greater muscle co-contraction in the pre-swing phase in patients with severe knee OA may stabilize the knee during propulsion. Our SHAP analysis also revealed that higher G activity and co-contraction of the TA–G in the swing phase are the most important EMG features for predicting WOMAC scores.

As knee OA severity progresses, several changes in temporospatial patterns may occur due to pain, impaired neuromuscular control, and altered biomechanics. As the G muscle flexes the knee, an increase in its activity decreases step and stride length^19^. Additionally, the step and stride lengths decreased, and cadence increased as OA severity increased. A higher cadence is associated with lower knee weight loading per step in patients with OA^52^.The present findings offer a framework to evaluate disease severity and devise knee OA treatment plans. Higher muscle co-activation serves as an objective measure to assess the knee OA severity. The differences in co-activation according to OA severity can be utilized to guide strategies for conservative treatment, such as unloader braces to reduce co-contraction^53^.

As a limitation, we did not enroll healthy patients as a control group. Moreover, various methods have been employed for PROMs-based categorization of knee OA severity, with no singular approach standardized in this field^37,38^. This variability in categorization could potentially influence study outcomes, suggesting a need for further investigation in future studies. Moreover, future studies are expected to be conducted with a large sample size of patients with knee OA. Furthermore, the paretic muscle location may vary among patients with knee OA. Since we didn't have maximum voluntary contraction (MVC) data available, we opted to normalize the EMG gait cycle by utilizing the maximum amplitude of each muscle^54–57^. However, it's important to note that results may differ depending on the normalization method, such as utilizing MVC, which warrants exploration in future prospectively collected datasets. The CCI measurements may vary depending on the antagonist and agonist muscle pairs and the normalization method of the muscle activity waveform. Furthermore, we plan to utilize a deep learning approach to estimate PROMs based on knee OA severity using muscle activity waveforms without performing feature extraction.

## Conclusion

We identified distinct neuromuscular features associated with knee OA, reflecting the functional limitations associated with knee osteoarthritis reported in WOMAC and VAS assessments. We also evaluated the effectiveness of a regression model to estimate PROMs of patients with advanced knee OA using normalized muscle activity and co-contraction features in the gait cycle. The identified muscle co-activation patterns and regression models may be utilized as objective candidate outcomes enhancing understanding the severity of knee OA and associated functional outcomes.

### Supplementary Information

Supplementary Figures.

## Data Availability

The datasets are available from the corresponding author upon reasonable request.
