# Supplementary material for: Estimation of patient-reported outcome measures based on features of knee joint muscle co-activation in advanced knee osteoarthritis
Source: Sci Rep. 2024 May 30;14:12428. doi: 10.1038/s41598-024-63266-7 (PMC11139965; doi:10.1038/s41598-024-63266-7)
Supplement: Supplementary file 1 — Supplementary Figures. [file 41598_2024_63266_MOESM1_ESM.docx]

**Estimation of Patient-Reported Outcome Measures based on Features of Knee Joint Muscle Co-activation in Advanced Knee Osteoarthritis**

Iqram Hussain, PhD ^1, 2, †^, Sung Eun Kim, MD ^3,†^, Chiheon Kwon, PhD ^4^, Seo Kyung Hoon *^5^*, Hee Chan Kim, PhD ^1,6,7^, Yunseo Ku, PhD ^4,5,*^, Du Hyun Ro, MD, PhD ^3,8,9,*^

^1^ *Institute of Medical and Biological Engineering, Medical Research Center, Seoul National University College of Medicine, Seoul 03080, South Korea*

*^2^ Department of Anesthesiology, Weill Cornell Medicine, Cornell University, New York, NY 10065, USA*

^3^ *Department of Orthopedic Surgery, Seoul National University Hospital, Seoul National University College of Medicine, Seoul 03080, South Korea*

^4^ *Medical Device Research Center, Department of Biomedical Research Institute, Chungnam National University Hospital, Daejeon 35015, Republic of Korea*

*^5^ Department of Biomedical Engineering, College of Medicine, Chungnam National University, Daejeon 35015, Republic of Korea*

*^6^ Department of Biomedical Engineering, Seoul National University College of Medicine, Seoul 03080, South Korea*

*^7^ Interdisciplinary Program in Bioengineering, Graduate School, Seoul National University, Seoul 08826, South Korea*

*^8^ CONNECTEVE Co., Ltd, Seoul 06224, South Korea*

*^9^ Innovative Medical Technology Research Institute, Seoul National University Hospital, Seoul 03080, South Korea*

**Nomenclature**

CCI: co-contraction index

RF: rectus femoris

MH: medial hamstring

TA: tibialis anterior

G: gastrocnemius

A: Average

MAX: maximum

MIN: minimum

ST: stance phase

SW: swing phase

T: time latency

AUC: area under the curve

VAS: visual analog scale

WOMAC: Western Ontario and McMaster Universities Osteoarthritis index

EMG: electromyography

Example of Feature Nomenclature:

G_SW_A: Average gastrocnemius amplitude in swing phase.

CCI_RF-G_ST_A: Average co-contraction of RF & G pairs in stance phase.


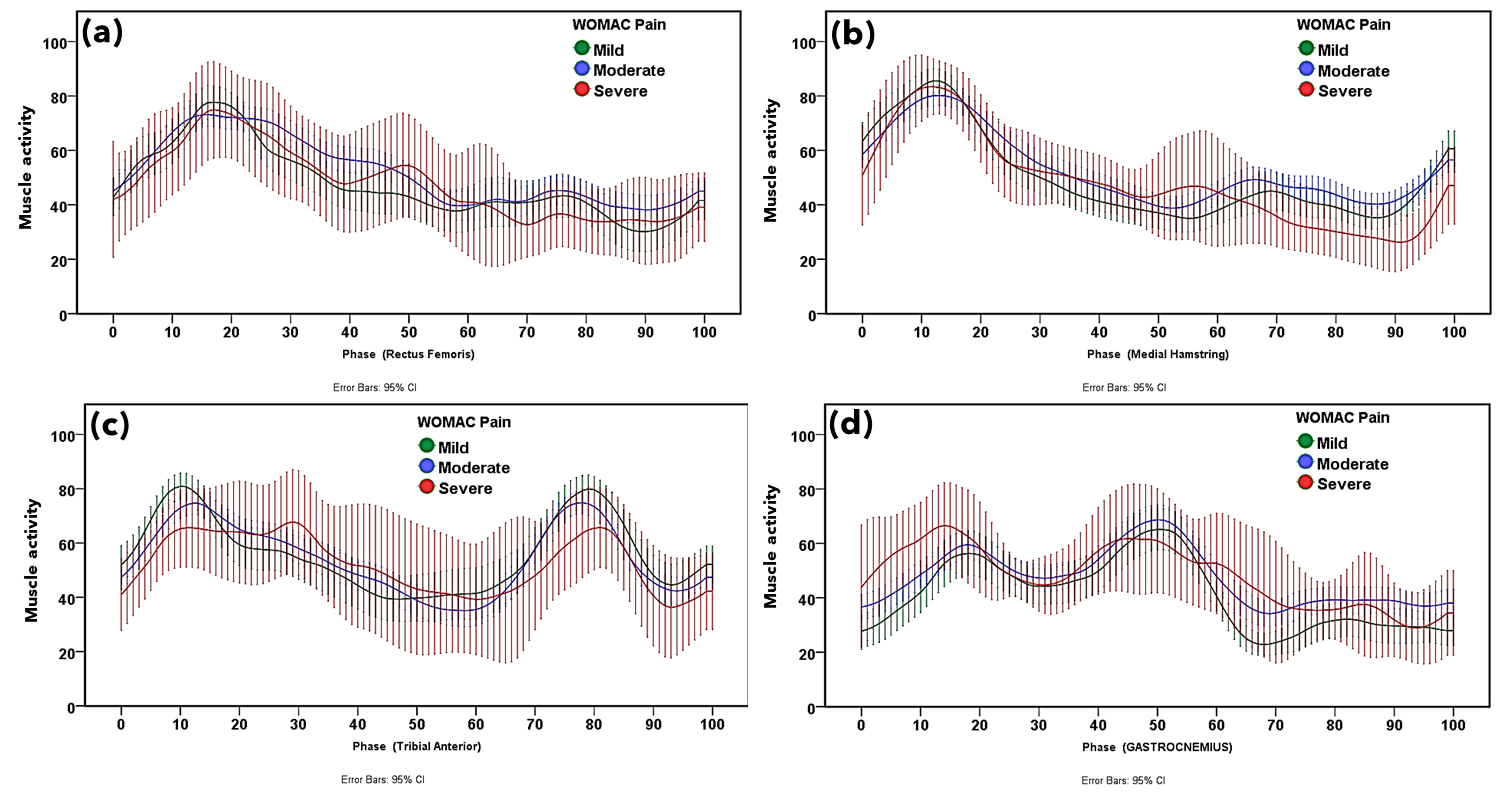


**Fig. S1.** Muscle activation pattern of the (a) rectus femoris, (b) medial hamstring, (c) tibialis anterior, and (d) gastrocnemius muscles in mild, moderate, and severe knee osteoarthritis according to the WOMAC pain subscore. The error bar shows 95% confidence interval.

**
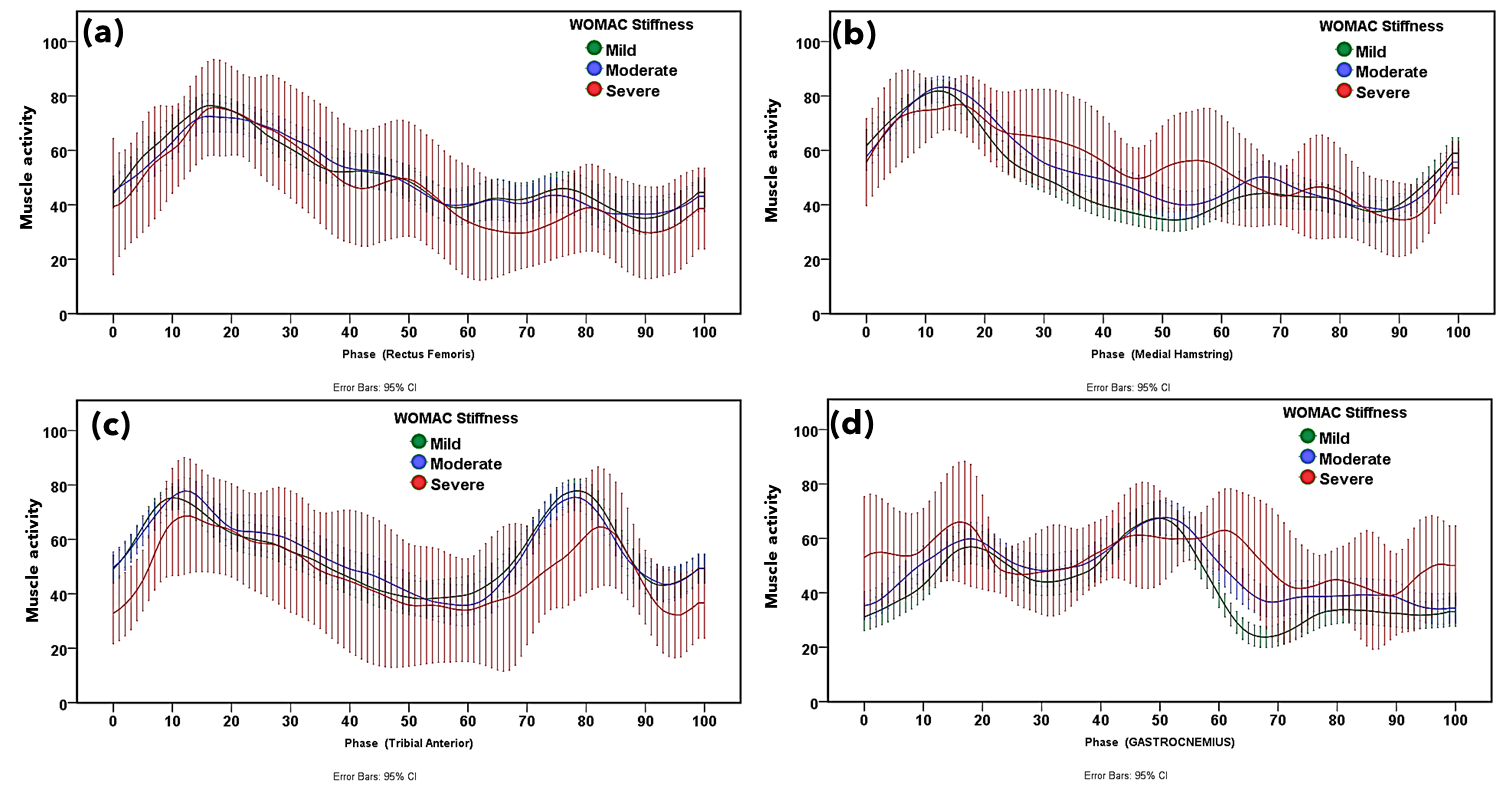
**

**Fig. S2.** Muscle activation pattern of (a) rectus femoris, (b) medial hamstring, (c) tibialis anterior, and (d) gastrocnemius in mild, moderate, and severe knee osteoarthritis according to the WOMAC stiffness subscore. The error bar shows 95% confidence interval.


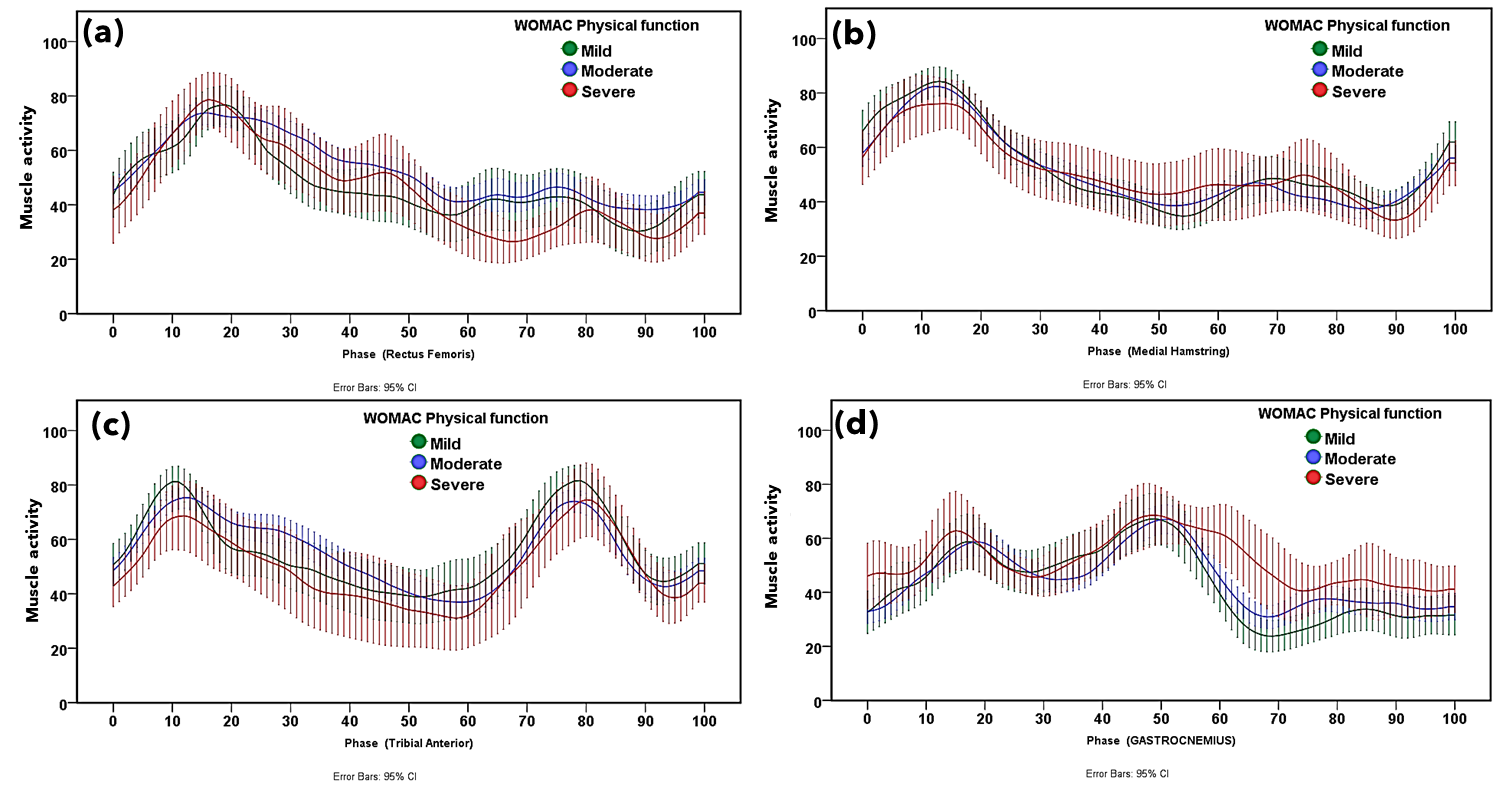


**Fig. S3.** Muscle activation pattern of (a) rectus femoris, (b) medial hamstring, (c) tibialis anterior, and (d) in mild, moderate, and severe knee osteoarthritis according to the WOMAC physical function subscore. The error bar shows 95% confidence interval.


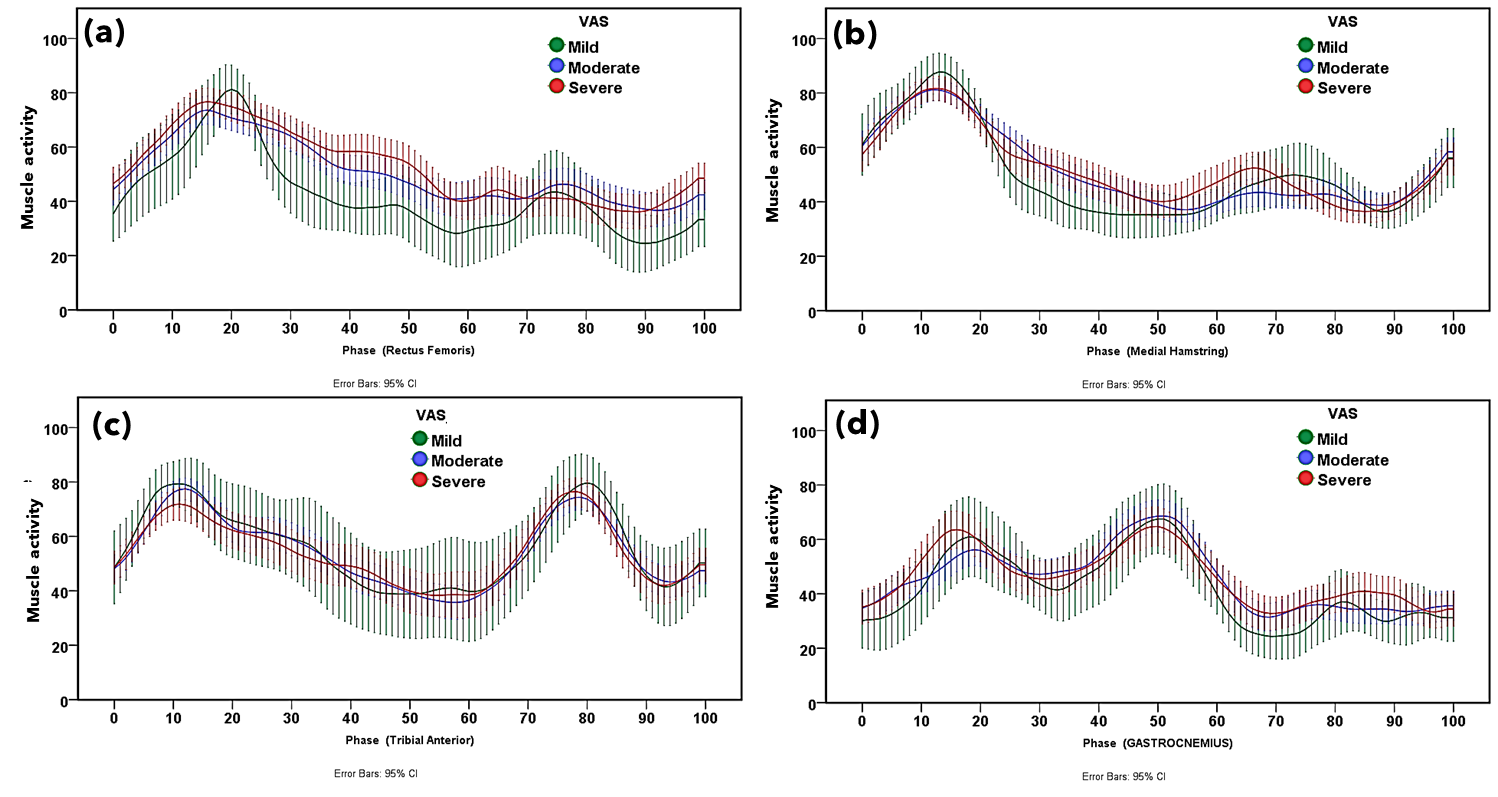


**Fig. S4.** Muscle activation pattern of (a) rectus femoris, (b) medial hamstring, (c) tibialis anterior, and (d) in mild, moderate, and severe knee osteoarthritis according to the visual analog pain score. The error bar shows 95% confidence interval.


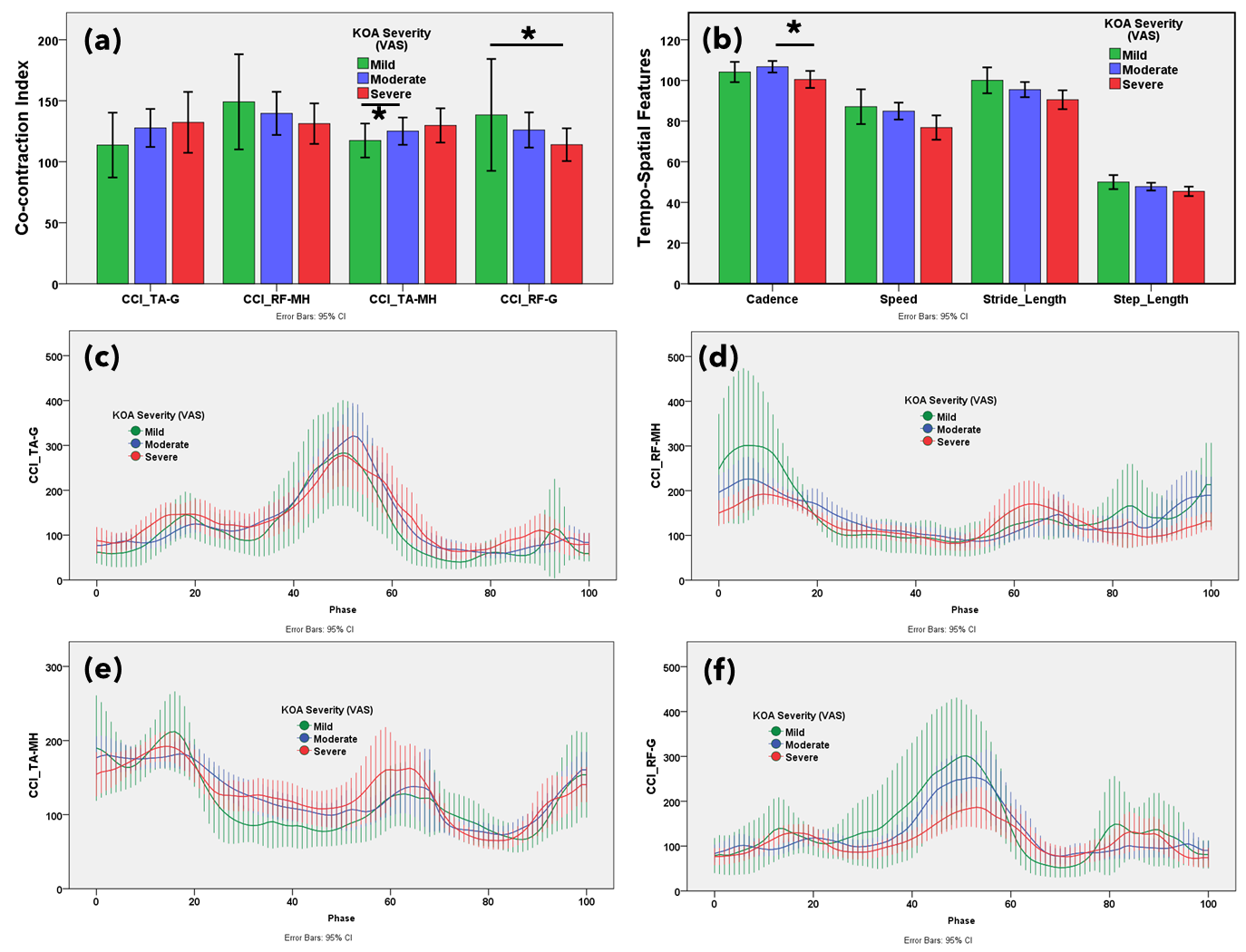


**Fig. S5.** (a) Statistical distribution of the co-contraction index for muscle pairs TA–G, RF–MH, TA–MH, and RF–G. (b) Temporospatial features in mild, moderate, and severe knee osteoarthritis based on the visual analog scale (VAS) for severity. Muscle co-activation patterns are depicted for (c) TA–G, (d) RF–MH, (e) TA–MH, and (f) RF–G pairs across different severity levels. Significance is denoted by ∗p < 0.05, indicating a significant difference. Error bars represent the 95% confidence interval.


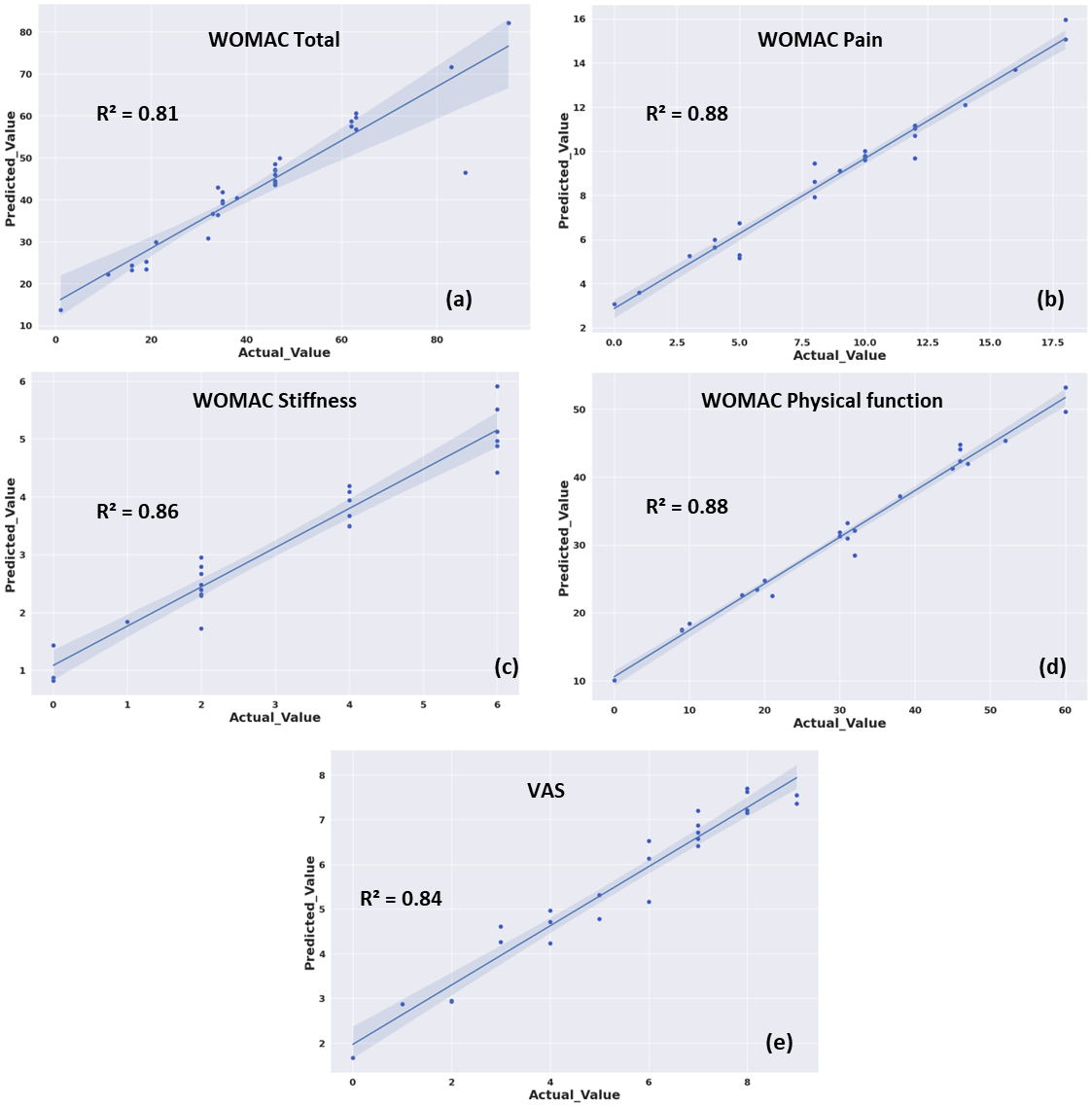


**Figure S6.** Regression Results for Estimating WOMAC Total Score Using a Random Forest Regression Algorithm Based on EMG Features with Testing Dataset. The scatter plot visualizes the prediction versus actual values for (a) WOMAC total, (b) WOMAC pain, (c) WOMAC stiffness, (d) WOMAC physical function, and (e) VAS scores. Key features include CCI (co-contraction index), RF (rectus femoris), MH (medial hamstring), TA (tibialis anterior), G (gastrocnemius), A (Average), MAX (maximum), MIN (minimum), ST (stance phase), SW (swing phase), T (time latency), AUC (area under the curve). Definitions: VAS - visual analog scale, WOMAC - Western Ontario and McMaster Universities Osteoarthritis index, EMG - electromyography.


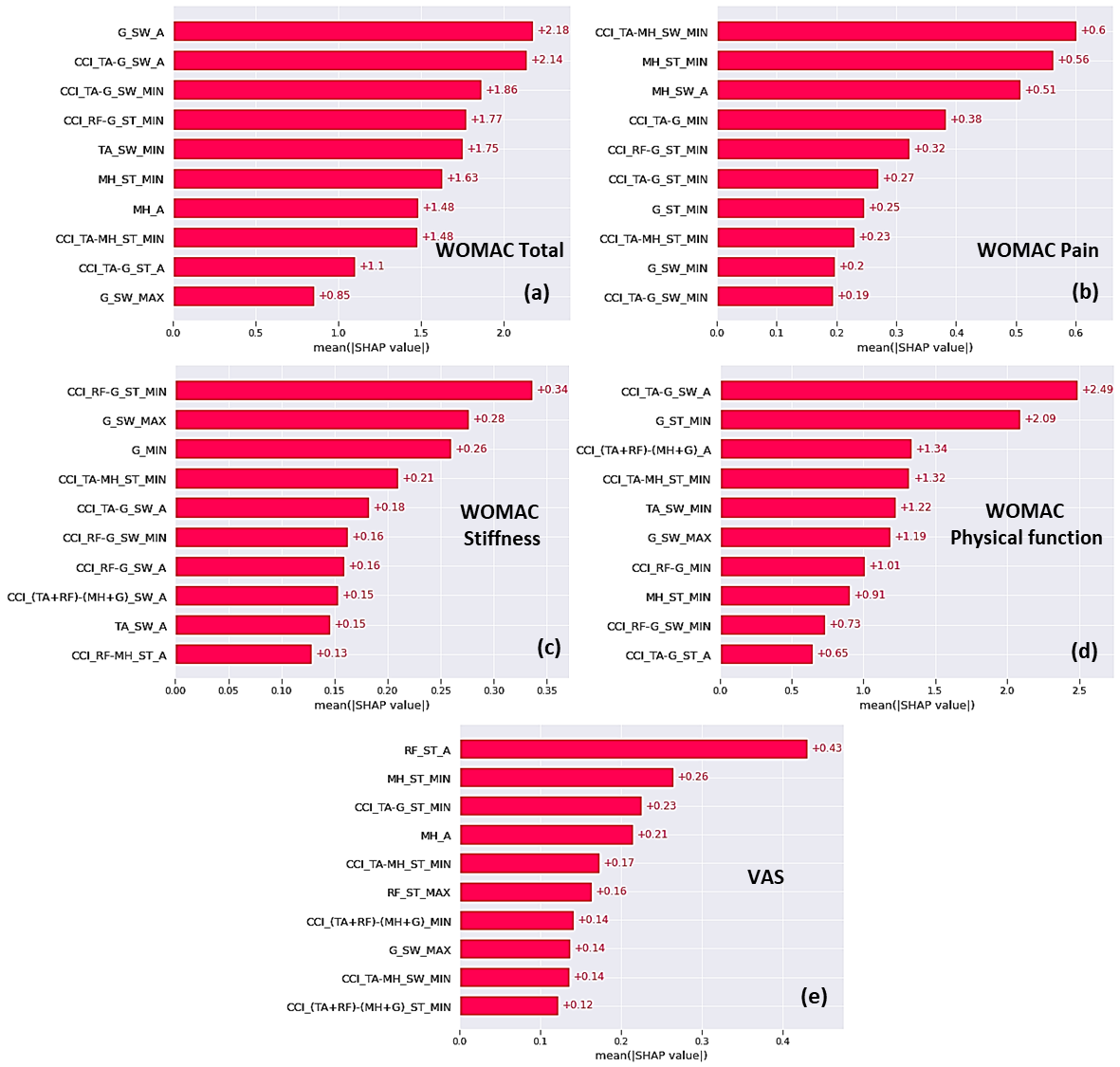


**Fig. S7.** SHAP feature importance plot of EMG features for estimating (a) WOMAC Total, (b) WOMAC pain, (c) WOMAC stiffness, (d) WOMAC physical function, and (e) VAS scores. Notable features include CCI (co-contraction index), RF (rectus femoris), MH (medial hamstring), TA (tibialis anterior), G (gastrocnemius), A (Average), MAX (maximum), MIN (minimum), ST (stance phase), SW (swing phase), T (time latency), AUC (area under the curve). Definitions: VAS - visual analog scale, WOMAC - Western Ontario and McMaster Universities Osteoarthritis index, EMG - electromyography.


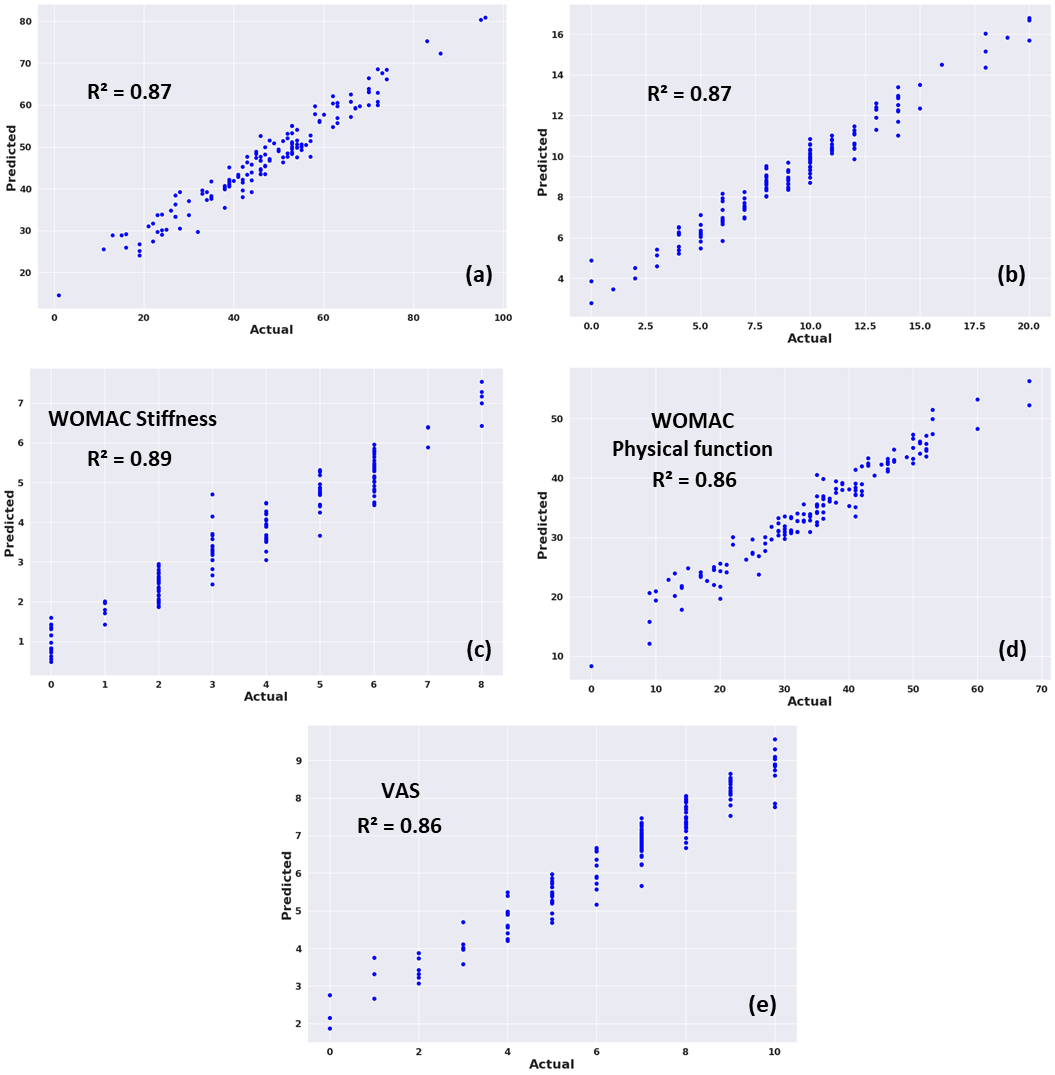


**Figure S8.** Performance outcomes of a machine-learning regression model assessing the severity of functional limitations via Patient-Reported Outcome Measures (PROMs), employing an RForest regression algorithm incorporating EMG and TS features. The figure depicts a scatter plot (blue) showcasing the machine learning predictions vs. actual PROMs score for (a) WOMAC total, (b) WOMAC pain, (c) WOMAC stiffness, (d) WOMAC physical function, and (e) VAS scores. CCI: co-contraction index, RF: rectus femoris, MH: medial hamstring, TA: tibialis anterior, G: gastrocnemius, A: Average, MAX: maximum, MIN: minimum, ST: stance phase, SW: swing phase, T: time latency, AUC: area under the curve; VAS, visual analog scale; WOMAC, Western Ontario and McMaster Universities Osteoarthritis index; EMG, electromyography; TS, temporospatial.


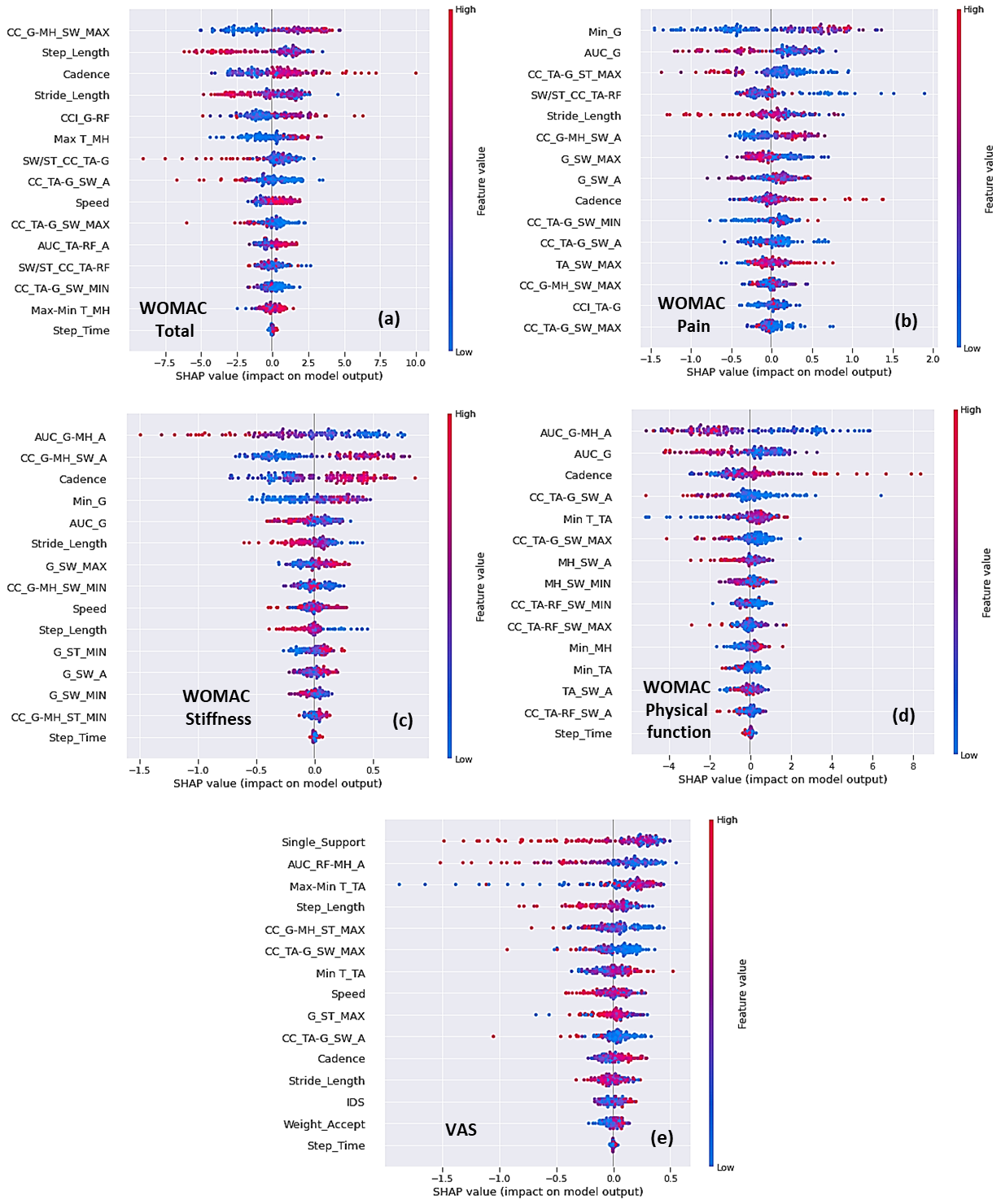


**Figure S9.** Interpreting the role of EMG and TS features in predicting knee OA severity, illustrated through the SHAP Summary plot, with specific attention to (a) WOMAC total, (b) WOMAC pain, (c) WOMAC stiffness, (d) WOMAC physical function, and (e) VAS scores, ranked by importance. CCI: CCI: co-contraction index, RF: rectus femoris, MH: medial hamstring, TA: tibialis anterior, G: gastrocnemius, A: Average, MAX: maximum, MIN: minimum, ST: stance phase, SW: swing phase, T: time latency, AUC: area under the curve; VAS: visual analog scale; WOMAC: Western Ontario and McMaster Universities Osteoarthritis index; EMG: electromyography; TS, temporospatial.
